# Functional division of labor in motility, lignocellulose digestion, and nitrogen metabolism revealed for the *Mixotricha paradoxa* holobiont

**DOI:** 10.1093/ismejo/wraf178

**Published:** 2025-08-20

**Authors:** Jieyang Fu, Yiting Liu, Takuya Yoshioka, Katsura Igai, Takako Mabuchi, Kumiko Kihara, Takumi Murakami, Nathan Lo, Moriya Ohkuma, Yuichi Hongoh

**Affiliations:** School of Life Science and Technology, Institute of Science Tokyo, 2-12-1 Ookayama, Meguro-ku, Tokyo 152-8550, Japan; School of Life Science and Technology, Institute of Science Tokyo, 2-12-1 Ookayama, Meguro-ku, Tokyo 152-8550, Japan; School of Life Science and Technology, Institute of Science Tokyo, 2-12-1 Ookayama, Meguro-ku, Tokyo 152-8550, Japan; School of Life Science and Technology, Institute of Science Tokyo, 2-12-1 Ookayama, Meguro-ku, Tokyo 152-8550, Japan; School of Life Science and Technology, Institute of Science Tokyo, 2-12-1 Ookayama, Meguro-ku, Tokyo 152-8550, Japan; School of Life Science and Technology, Institute of Science Tokyo, 2-12-1 Ookayama, Meguro-ku, Tokyo 152-8550, Japan; School of Life Science and Technology, Institute of Science Tokyo, 2-12-1 Ookayama, Meguro-ku, Tokyo 152-8550, Japan; School of Life and Environmental Sciences, University of Sydney, Corner of Parramatta and City roads, Sydney, NSW 2006, Australia; Japan Collection of Microorganisms, RIKEN BioResource Research Center, 3-1-1 Koyadai, Tsukuba, Ibaraki 305-0074, Japan; School of Life Science and Technology, Institute of Science Tokyo, 2-12-1 Ookayama, Meguro-ku, Tokyo 152-8550, Japan; Japan Collection of Microorganisms, RIKEN BioResource Research Center, 3-1-1 Koyadai, Tsukuba, Ibaraki 305-0074, Japan

**Keywords:** symbiosis, termite, holobiont, spirochete, protozoa

## Abstract

*Mixotricha paradoxa* is a large cellulolytic flagellate present in the hindgut of the termite *Mastotermes darwiniensis*. This parabasalid flagellate is unique in its reliance on ectosymbiotic spirochetes for motility. We analyzed the transcriptome of *M. paradoxa* and the genomes of the ectosymbiotic spirochete *Propulsinema mixotrichae* (“*Treponematales*”), the rod-shaped ectosymbiont *Synergitannerella mixotrichae* (*Bacteroidales*), and the endosymbiont *Endomicrobiellum mixotrichae* (*Endomicrobiales*), all of which are obligately associated with *M. paradoxa* and were taxonomically described in this study. *Mixotricha paradoxa* highly expressed genes for diverse glycoside hydrolases (GHs) and likely ferments sugars to H_2_, CO_2_, acetate, ethanol, and glycerol. Similar to the case for parasitic parabasalids such as *Trichomonas vaginalis*, transcripts for biosynthesis of nucleotides and many amino acids were not detected in our analyses of *M. paradoxa*. *Propulsinema mixotrichae* possesses genes encoding proteins for the assembly of flagella and for those in pathways associated with chemotaxis and dinitrogen fixation. Such genes are absent in *S. mixotrichae*, which instead possesses numerous genes encoding glycoside hydrolase enzymes, which are largely complementary to the glycoside hydrolase repertoire of *M. paradoxa*. *Endomicrobiellum mixotrichae* appears to provide nucleotides and nine amino acids to its host, which in turn likely supplies three amino acids, including tryptophan, to *E. mixotrichae*. Because bacterial cells, in addition to wood particles, were observed in food vacuoles of *M. paradoxa*, these ecto- and endosymbionts may be digested by the flagellate host. Overall, the distinct roles of each symbiont highlight the efficient functional division of labor that has evolved in this holobiont.

## Introduction

Termites are eusocial insects widespread in temperate to tropical regions and play a critical role in lignocellulose decomposition, in concert with their hindgut microbiota [1, 2]. With the exception of the family Termitidae, termites harbor dense communities of flagellated protists in their guts—belonging to either the phylum *Parabasalia* or the order *Oxymonadida* in the phylum *Preaxostyla*—in addition to bacteria, archaea, and viruses [2, 3]. These gut flagellates play a primary role in lignocellulose hydrolysis, oxidizing sugars to acetate, which serves as a carbon and energy source for the termite host [2]. Diverse gut prokaryotes also contribute to lignocellulose digestion [4–8] and other functions such as nitrogen fixation and recycling [9–14], upgrading of nitrogen compounds [10, 12–16], removal of hydrogen [10, 12, 13, 17–19], and CO_2_-reductive acetogenesis [12, 13, 20, 21]. A portion of these gut prokaryotes specifically inhabit the surface [4, 11, 14, 17, 18, 22, 23], cytoplasm [10, 12, 13, 15, 16, 19, 24, 25], and nuclei [26] of the gut flagellates.

**Figure 1:**
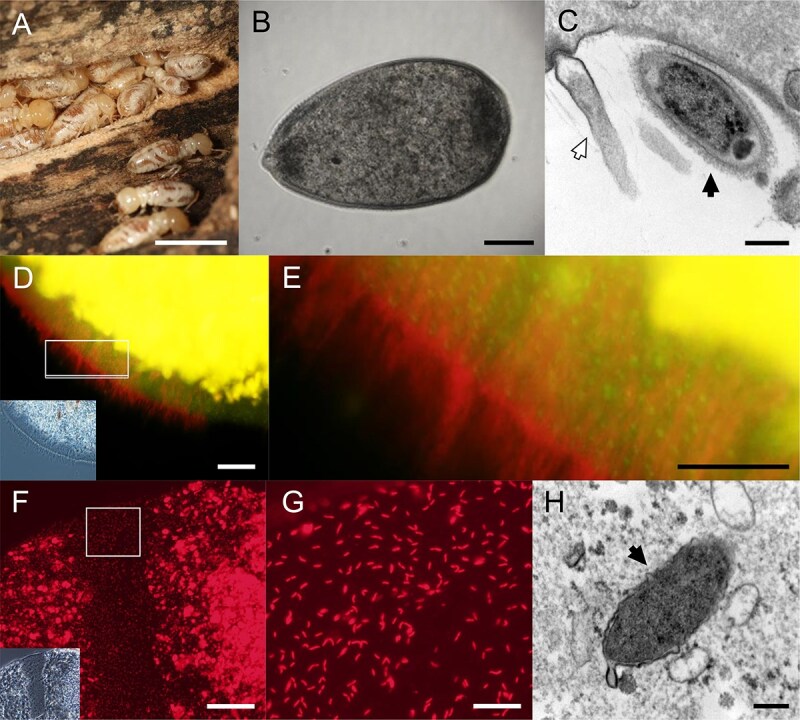
*Mastotermes darwiniensis*, *Mixotricha paradoxa*, and bacterial ecto- and endosymbionts of *M. *p*aradoxa*. (A) *Mastotermes darwiniensis*. (B) Differential interference contrast-image of *M. paradoxa*. (C) Transmission electron micrograph (TEM) of *Propulsinema mixotrichae* (white arrow) and *Synergitannerella mixotrichae* (black arrow) attached to a “bracket” structure. (D) Detection of *P. mixotrichae* (spiral-shaped) and *S. mixotrichae* (rods) by fluorescence *in situ* hybridization (FISH) using probes MdMp-014-133 (Texas red-labeled, red) and MdMp-bact197 (6FAM-labeled, green) specific to each bacterium (Table S1). The epifluorescent images were merged. Amorphous yellow area is autofluorescence emitted from wood fragments phagocytosed by *M. paradoxa.* Corresponding phase-contrast image is shown as inset. (E) Magnified image of the area indicated in panel D. (F) Detection of *Endomicrobiellum mixotrichae* (rods) by FISH using the specific probe MdMp-027-644 (Texas red-labeled, red) (Table S1). Amorphous area is autofluorescence from wood fragments. Corresponding phase-contrast image is shown as inset. (G) Magnified image of the area indicated in panel F. (H) TEM of a putative cell of *E. mixotrichae* (black arrow) in the host cytoplasm. Bars: (A) 1 cm; (B) 120 μm; (C, H) 200 nm; (D) 20 μm; (E, G) 10 μm; (F) 50 μm.

*Mixotricha paradoxa* is a large parabasalid flagellate with ca. 300–500 μm in length exclusively found in the hindgut of the termite *Mastotermes darwiniensis* [27–29] (Fig. 1A and B). The cell surface of *M. paradoxa*, except for the posterior region, is densely covered with spirochetes and rod-shaped bacteria, both of which are attached to regularly arranged structures known as “brackets” [30–33] (Fig. 1C). Unlike other flagellates, *M. paradoxa* is propelled exclusively by the locomotory waves of the ectosymbiotic spirochetes (Supplementary Videos S1 and S2), whereas the four flagella of the host serve only to steer the cell into a new direction [30, 31, 33]. Previous studies indicated that the ectosymbiotic spirochetes comprised three 16S rRNA phylotypes (mp1, mp3, and mpsp15) and that mpsp15 was predominant and localized at the anterior side of the brackets, whereas mp1 and mp3 were localized only at a restricted posterior portion of the host cell surface [32, 34]. A previous study [31] proposed that the physical contact between the regularly arranged short spirochetes (which obviously correspond to mpsp15 [32]) is associated with the synchronized waves of the spirochetes.

In addition to the spirochetes, a rod-shaped bacterium, “B6”, belonging to the order *Bacteroidales* [32, 34], is always localized at the bottom of the brackets [30, 32, 33] (Fig. 1C). *Mixotricha paradoxa* also harbors the 16S rRNA phylotype MdMp-027 of the genus *Endomicrobiellum* (order *Endomicrobiales*) intracellularly [35]. However, as these microbes are uncultivable, their interrelationships remain unexplored.

The concept of the “holobiont”—a host and its associated microorganisms functioning as an integrated biological entity—has reshaped our understanding of symbiosis, evolution, and ecological adaptation [36]. Holobionts can exhibit functional specialization and division of labor among symbiotic partners. For example, in a sharpshooter, the dual bacterial endosymbionts *Sulcia muelleri* and *Baumannia cicadellinicola* exhibit strong metabolic complementarity with each other and the host; *S. muelleri* synthesizes essential amino acids, whereas *B. cicadellinicola* produces cofactors, jointly compensating for the nutrient-poor plant sap diet [37]. An allocation of functions also occurs in the cnidarian–dinoflagellate holobionts, where exchanges of carbon and nitrogen compounds between partners are essential for the holobiont survival [38]. Similar evolutionary dynamics promoting metabolic specialization and interdependence among symbionts may also operate within the *M. paradoxa* holobiont.

In this study, we investigate the *M. paradoxa* hologenome by analyzing transcriptomes of the host *M. paradoxa* and (near-)complete genomes of its three obligate bacterial symbionts, in order to uncover the division of labor that has evolved within this holobiont. Our findings reveal how coevolution has driven functional specialization among partners and contributed to the evolutionary success of this holobiont.

Here we describe the three primary bacterial symbionts of *M. paradoxa* as *Propulsinema mixotrichae* (corresponding to mpsp15), *Synergitannerella mixotrichae* (B6), and *Endomicrobiellum mixotrichae* (MdMp-027) under the “Code of Nomenclature of Prokaryotes Described from Sequence Data” (SeqCode) [39].

## Materials and methods

### Termites and flagellates

*Mastotermes darwiniensis* (family Mastotermitidae) were collected at Darwin, Australia and kept in plastic containers with infested wood fragments in the laboratory. The entire gut was removed from worker termites, and the gut contents were suspended in sterile Trager’s solution U [40]. Single cells of *M. paradoxa* were isolated using a Leica AM6000 inverted micromanipulation microscope system and were washed several times in droplets of solution U.

### Phylogenetic analysis, fluorescence *in situ* hybridization, and transmission electron microscopy

Reference sequences for the phylogenetic analyses of 16S rRNA, NifH, and TrpB were retrieved as described in the Supplementary Methods. Maximum likelihood trees were constructed using IQ-TREE v1.6.12 with 1000 ultrafast bootstrap and SH-aLRT test replicates [41].

Oligonucleotide probes specific to the respective 16S rRNA genes were designed using ARB [42] (Table S1). Fluorescence *in situ* hybridization (FISH) was performed as described previously [22, 43]. Specimens were observed under an Olympus BX51 epifluorescence microscope. Transmission electron microscopy (TEM) of *M. paradoxa* was performed as described previously [26] and observed under an H-7500 transmission electron microscope (Hitachi).

### Preparation of DNA for genome sequencing

To enrich ectosymbionts, a single *M. paradoxa* cell was dissected with a microblade [17] into two parts, and only the anterior cell membrane portion without the host nucleus was collected. Eighteen such “anterior surface” samples were prepared, and each was subjected to whole-genome amplification (WGA) using EquiPhi29 DNA polymerase (ThermoFisher Scientific) (Table S2). After a high-quality genome of *P. mixotrichae* was obtained from one of these samples as below, we attempted to prepare a sample mainly containing the genomic DNA of *S. mixotrichae* by removing *P. mixotrichae* cells*.* Assuming that spirochetal cells are more susceptible to detergents than the cells of *Bacteroidales*, we treated additional samples with 0.5% Tween 20 and 0.1 U/μL DNase I for 20–35 min at room temperature. As a result, one of the four samples generated genome fragments of *S. mixotrichae* almost exclusively without genomic fragments of spirochetes. Cells of endosymbiotic bacteria were collected from single *M. paradoxa* cells with a fine glass capillary after being treated with 0.1% Tween 20. The prepared samples were summarized in Tables S3 and S4. Details are described in the Supplementary Methods.

### Genome sequencing and selection of samples

Paired-end sequencing libraries were prepared from the WGA samples and sequenced on an MiSeq System (Illumina) using the MiSeq Reagent Kit V3 (600 cycles). The samples were compared with respect to the completeness and contamination rate of genomes, which were predicted using CheckM v1.1.3 [44]. Based on these comparisons, we selected three samples, which generated the highest-quality genomes of *P. mixotrichae*, *S. mixotrichae*, and *E. mixotrichae*, respectively (Table S3). No high-quality genomes of other prokaryotic species were recovered (Table S4). The three samples were subjected to a second-round WGA (Table S5), and paired-end sequencing was performed as described above. In addition, long reads (2–10 kb) were generated from the second-round WGA samples on a MinION platform (Oxford Nanopore Technologies). Details, including quality trimming of reads, are described in the Supplementary Methods.

### Genome assembly and binning

The quality-trimmed MiSeq reads were assembled into contigs using SPAdes v3.15.0 with the default --sc mode [45]. Contigs were binned based on tetranucleotide frequency using MyCC_2017 [46]. The contigs of *P. mixotrichae* and *S. mixotrichae* were scaffolded with the quality-trimmed MinION reads using SLR [47]. For the sample containing *E. mixotricha*e, the quality-trimmed MinION reads were assembled using Flye v2.8 [48], resulting in a circular contig, which was polished using the quality-trimmed reads. Detailed methods, including decontamination of contigs and verification of ambiguous regions, are described in the Supplementary Methods.

### Genome annotation

The genomes were subjected to gene prediction and annotation using DFAST [49] and RAST [50]. Gene loci and annotations were manually curated based on the results of BLASTp [51] searches of the NCBI nonredundant (nr) protein database. Only sequences of ≥100 amino acids were considered putative protein-coding sequences (CDSs) unless shorter ones exhibited significant homology to database sequences. Pseudogenes were identified manually [15]. Metabolic pathways were inferred with the KEGG automatic annotation server (KAAS) [52] and KEGG Mapper [53]. Genes for carbohydrate-active enzymes (CAZymes) were predicted using dbCAN v4.0.0 [54]. Secretion system-related genes were predicted using TXSScan [55]. The substrates of F-type and V-type ATPases were predicted according to a previously study [56]. Genes and pseudogenes were classified into clusters of orthologous genes (COG) functional categories using RPS-BLAST v2.2.26 [57].

### Phylogenomics and comparative genomics

For phylogenomic analysis, reference sequences were retrieved using GTDB-Tk v2.1.0 [58] (see Supplementary Methods), with >80% completeness and <5% contamination rate, predicted using CheckM2 [59], being retained. Average nucleotide identity (ANI) was calculated using FastANI v1.33 [60]. Genomes with ANI ≥95% were clustered, and a representative sequence with higher completeness was retained (Tables S6 and S7). Conserved single-copy marker genes were extracted, concatenated, and aligned as described in the Supplementary Methods. Maximum-likelihood trees were constructed using IQ-TREE v1.6.12 as above.

### Library preparation for RNA-seq

A single or three cells of *M. paradoxa* were collected as above in separate PCR tubes containing RNAlater Solution (Qiagen) and stored at −80°C. RNA was extracted using the Quick-RNA Fungal/Bacterial Microprep Kit (Zymo Research) and subjected to cDNA synthesis and amplification using the SMART-Seq v4 Ultra Low Input RNA Kit (Clontech). Fragments of 550–700 bp were used for the preparation of sequencing libraries (see Supplementary Methods).

### Transcriptome sequencing and assembly

*Mixotricha paradoxa* cDNA libraries were sequenced on the MiSeq System as above. The quality-trimmed reads were assembled using Trinity v2.6.6 [61]. The contigs were filtered and decontaminated using the metagenomic reads of the associated bacteria (Table S4) and then used for downstream analyses. Transcriptome completeness was predicted by BUSCO v4.0.6 [62] with the dataset “eukaryota_odb10.2019-11-2”. Transcripts per million (TPM) of the filtered contigs were calculated using salmon v1.7.0 [63]. Metabolic pathways were reconstructed using the KAAS and KEGG Mapper. Details, including prediction of open reading frames and functional annotation, are described in the Supplementary Methods.

## Results

### Localization and morphology of symbiotic bacteria

Cells of the three bacterial symbionts were detected by FISH targeting their 16S rRNA with specific probes (Table S1). We confirmed that *P. mixotrichae* and *S. mixotrichae* are obligate ectosymbionts covering a large part of the host cell surface (Figs. 1D, E, and S1) as reported previously [30, 32]. *Endomicrobiellum mixotrichae* cells were detected as rods that appeared to be evenly distributed throughout the host cytoplasm (Fig. 1F, G). TEM revealed a regular arrangement of *P. mixotrichae* and *S. mixotrichae* on the brackets, with the anterior end of *P. mixotrichae* cells being inserted into the host cell surface (Figs. 1C and S2A) whereas *S. mixotrichae* cells being attached with a fluffy surface layer (Fig. 1C) as reported previously [30, 32]. Rod-shaped cells putatively of *E. mixotrichae* were observed in the cytoplasm of *M. paradoxa* by TEM (Fig. 1H). Food vacuole-like structures of *M. paradoxa* that contained bacterial cells, including spirochetes and rods resembling *E. mixotrichae*, were also observed (Fig. S2B–E).

### Phylogenetic positions of symbiotic bacteria

The 16S rRNA gene tree indicated that *P. mixotrichae* belongs to the family *Breznakiellaceae* [64] and clustered with other spirochetes derived from termite guts (Fig. S3) [22, 32]. *Synergitannerella mixotrichae* was assigned to the family *Tannerellaceae* and clustered with other rod-shaped ectosymbionts of parabasalids in the guts of termites and the wood-feeding cockroach *Cryptocercus punctulatus* (Fig. S4) [65–67]. The 16S rRNA gene sequence of *E. mixotrichae* was identical with that of Md513_bin60, a metagenome-assembled genome (MAG) with 63.7% completeness recovered from the gut of *M. darwiniensis* [68]. Md513_bin60 belongs to the genus *Endomicrobiellum*, which exclusively comprises sequences from the guts of protist-dependent termites [68].

### *Mixotricha paradoxa* expresses cellulolytic enzymes but lacks many biosynthetic pathways

Transcriptomes were obtained from a single cell or three cells of *M. paradoxa* (Table S8). The estimated completeness of gene repertoires was ca. 35% in the three-cell sample, which is similar to that found in a previous study of parabasalids in the termite gut (Fig. S5) [69]. Considering that high-quality genomes of pathogenic parabasalids exhibit only up to 60% completeness in the BUSCO estimation (Fig. S5) [70], our transcriptome data possibly cover ca. 60% (i.e. 35% of 60%) of the *M. paradoxa* genome. We are unable to rule out the presence of contaminating transcripts from other flagellate species, however we expect these to be minimal [69].

**Figure 2:**
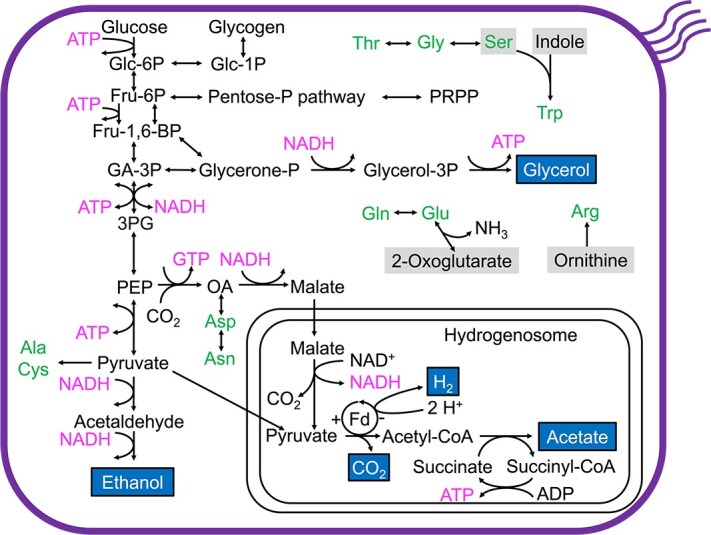
Predicted metabolic pathways of *Mixotricha paradoxa* based on transcriptome. Putative metabolic end products (e.g., acetate) are highlighted and framed. Shaded substrates (e.g., indole) are required to be supplemented by symbionts or from other sources. Abbreviations: Glc-1P, glucose-1P; Glc-6P, glucose-6P; Fru-6P, fructose-6P; Fru-1,6-BP, fructose-1,6P_2;_ GA-3P, glyceraldehyde-3P; 3PG, glycerate-3P; PEP, phosphoenolpyruvate; OA, oxaloacetate; Fd, ferredoxin.

The predicted basic metabolism of *M. paradoxa* closely resembles those of the pathogenic parabasalids *Trichomonas vaginalis* and *Tritrichomonas foetus* [71, 72]. Pathways for glycolysis and gluconeogenesis are likely complete (Fig. 2). Expression of the glycerol kinase [EC:2.7.1.30] gene suggested that *M. paradoxa* has capacity to convert glycerol-3-phosphate to glycerol, as reported previously in the above two trichomonads [72]. The presence of aldehyde dehydrogenase and alcohol dehydrogenase indicated an ability to produce ethanol as a fermentation product. Based on sequence similarities to the hydrogenosomal proteins of *Trichomonas vaginalis* [71, 72], we predicted that, in the hydrogenosomes of *M. paradoxa*, hydrogen is produced using Fe-hydrogenase, and acetyl-CoA is converted to acetate by the action of acetate: succinate CoA-transferase. Transcripts of a gene homologous to H_2_-oxidizing, cytoplasmic Fe-hydrogenase of *Trichomonas vaginalis* [73] were also detected.

Biosynthetic pathways for most cofactors and nine proteinogenic amino acids were not detected (Fig. 2). Transcripts for tryptophan synthase TrpB were identified, which potentially enable tryptophan synthesis from indole and serine [74]. TrpB of *M. paradoxa* is phylogenetically closest to that of the parabasalid *Pseudotrichonympha grassii* (Fig. S6). Although our transcriptome analyses indicated that 5-phospho-α-D-ribose 1-diphosphate (PRPP) can be synthesized (Fig. 2), gene transcripts required for de novo nucleotide synthesis were not detected. Comparative transcriptomic analysis among parabasalid flagellates revealed that *M. paradoxa* exhibited relatively low expression levels of metabolism-related pathways and high expression levels of cellular processes, mainly due to the higher expression level of actin. *Mixotricha paradoxa* showed low expression levels of genes related to “cilium-dependent cell motility” (Fig. S7).

*Mixotricha paradoxa* expressed genes of 29 GH families. The most highly expressed families were GH10, GH7, and GH45, which typically encode xylanase, cellobiohydrolase, and endoglucanase, respectively (Fig. S8; Table S7). Genes for β-xylosidase, β-mannanase, and α-glucuronidase were also expressed; thus, *M. paradoxa* likely hydrolyzes cellulose and the main components of hemicellulose.

### *Propulsinema mixotrichae* (“*Treponematales*”) possesses genes for motility and nitrogen fixation

**Figure 3:**
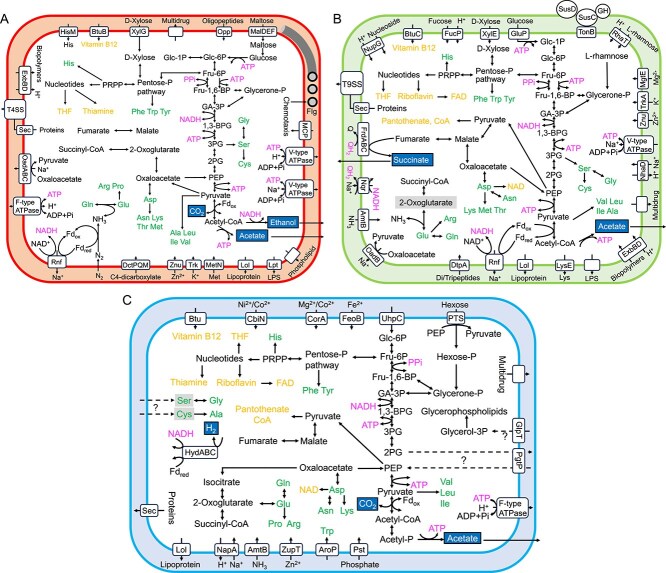
Predicted metabolic pathways of bacterial symbionts of *Mixotricha paradoxa*. (A) *Propulsinema mixotrichae*. (B) *Synergitannerella mixotrichae*. (C) *Endomicrobiellum mixotrichae*. Putative fermentation products (e.g., acetate) are highlighted and framed. Shaded substrates (e.g., 2-oxoglutarate) are required to be supplemented by the hosts, other symbionts, and/or other sources. Abbreviations: Glc-1P, glucose-1P; Glc-6P, glucose-6P; Fru-6P, fructose-6P; Fru-1,6-BP, fructose-1,6P_2_; GA-3P, glyceraldehyde-3P; 1,3-BPG, glycerate-1,3P_2_; 3PG, glycerate-3P; 2PG, glycerate-2P; PEP, phosphoenolpyruvate; Fd_ox/red_, ferredoxin oxidized/reduced; Flg, flagella.

The genome size of *P. mixotrichae* was ca. 3.9 Mbp (Table 1), which is larger than those of many other species of *Breznakiellaceae*, including free-swimming species (Fig. S9A; Table S6). The genome encodes the complete pathway for glycolysis/gluconeogenesis, and the main carbon sources are likely monosaccharides and disaccharides such as D-xylose and maltose (Fig. 3A). ATP can be generated via glycolysis and fermentation to acetate, ethanol, and CO_2_, and H^+^/Na^+^ membrane potential-mediated energy conservation is likely achieved by the action of the Rnf complex and F-type and/or V-type ATPase (Fig. 3A). Genes encoding hydrogenase were not identified.

**Table 1:** General genome features of symbiotic bacteria associated with *M. paradoxa*.

Species	Order	Lifestyle	Contigs (>1000 bp)	Total length (bp)	GC content (%)	Complete-ness (%)^*^	Contamination rate (%)^*^	CDS	Coding density	Pseudo-gene^**^	rRNA gene	tRNA gene
*Propulsinema mixotrichae*	*Treponematales*	Ectosymbiont	153	3 886 429	59.4	95.7	2.3	3061	0.69	202	3	56
*Synergitannerella mixotrichae*	*Bacteroidales*	Ectosymbiont	268	3 157 688	48.4	99.2	4.1	1991	0.76	100	3	53
*Endomicrobiellum mixotricha*e	*Endomicrobiales*	Endosymbiont	1 (circular)	1 154 836	34.8	100	0.1	747	0.64	70	3	45

^^*^^Estimated by CheckM2.

^^**^^Manually identified.

Nucleotides and all proteinogenic amino acids appear to be de novo synthesized, and nitrogen can be provided via dinitrogen fixation. Two nitrogenase reductase gene homologs (*nifH1* and *nifH2*) assigned to the NifH group II were identified (Figs. S10– S12; Tables S3 and S9; see Supplementary Results) [75]. The genome encodes almost the entire flagellar and chemotaxis assemblies. Components of the Sec system and the type IV secretion system (T4SS) are also present (see Supplementary Results). The genome encodes leucine-rich repeat proteins [76], fibronectin (Fn) III-type domain proteins [77], and ankyrin repeat domain proteins [78]. The T4SS and these proteins are possibly associated with adhesion to the host cell surface.

### *Synergitannerella mixotrichae* (*Bacteroidales*) possesses diverse hemicellulolytic enzyme genes

The genome size of *S. mixotrichae* was ca. 3.2 Mbp (Table 1), which is similar to those of other ectosymbiotic or free-living species of *Bacteroidales* (Fig. S9B). The genome encodes the complete pathway for glycolysis/gluconeogenesis, and the main carbon sources are likely diverse plant-derived sugars. The genome encodes 42 GH genes across 20 families, 27 of which contain a signal peptide sequence (Table S10). No GH genes involved in cellulose hydrolysis were identified except for β-glucosidase, whereas various GH genes for the hydrolysis of hemicellulose and pectin were found. For example, genes coding for xylanase, α-L-arabinofuranosidase, α-1,2-mannosidase, α-L-rhamnosidase, polygalacturonase, glucuronyl hydrolase, and α-L-fucosidase were identified (Tables S10 and S11). The genome encodes 25 paralogs for oligosaccharide transporter SusC and 30 paralogs for glycan-binding protein SusD (Fig. 3B), both of which are characteristic in plant fiber-degrading *Bacteroidota* [79].

*Synergitannerella mixotrichae* can generate ATP via glycolysis and fermentation to acetate and succinate. It likely conserves energy using the Rnf complex and V-type ATPase. No genes responsible for nitrogen fixation were identified; *S. mixotrichae* can use ammonium and di/tripeptides as its main nitrogen sources. The bacterium can synthesize nucleotides, several cofactors, and all proteinogenic amino acids except for proline (Fig. 3B).

A complete type IX secretion system (T9SS) was identified (Fig. S13), which is characteristic to *Bacteroidota* and aids both gliding motility and protein secretion [80, 81]. Because the key components for gliding motility, SprB and RemA [82, 83], were not found, the bacterium is likely non motile. Adhesin [84], chitinase [85], and cellulase [86] can reportedly be secreted through T9SS. In *S. mixotrichae*, 51 out of 56 immunoglobulin-like domain-containing proteins, predicted to be adhesins, contained both a signal peptide for the Sec system and conserved motifs in the C-terminal domain recognized by T9SS (Fig. S14) [81]. In contrast, the GH family proteins lack the known motifs for T9SS, even though many possess signal peptide sequences for the Sec system.

### *Endomicrobiellum mixotricha*e (*Endomicrobiales*) has a compact genome and potential dependence on host metabolites

The complete, circular chromosome of *E. mixotrichae* was obtained (Table 1), which is similar in size (1.15 Mbp) to those of other *Endomicrobiellum* species [68]. Although it likely imports glucose-6-phosphate (G6P) via a UhpC-homolog transporter as predicted in other *Endomicrobiellum* species [15, 68], its glycolytic pathway is disrupted by the absence of enolase (Fig. 3C). In addition, the genome lacks the gene for glycerol-3-phosphate dehydrogenase; therefore, the bacterium cannot de novo synthesize glycerophospholipids from glycerone phosphate. Two additional UhpC homologs may transport phosphoenolpyruvate (PEP) and glycerol-3-phosphate, respectively, thereby complementing the metabolic pathways (Fig. S15; see Discussion).

The bacterium can additionally import hexose as a carbon source via a PEP-dependent phosphotransferase system (PTS). However, the disruption of the glycolytic pathway has likely made it difficult for the bacterium to directly use sugars as energy sources. If PEP can be imported as hypothesized above, PEP should be the main energy source to generate ATP (Fig. 3C). The fermentation products are estimated to be acetate, CO_2_ and H_2_. *Endomicrobiellum mixotricha*e encodes biosynthetic pathways for 15 amino acids. Among them, the biosyntheses of glycine and alanine require import of serine and cysteine as substrates, respectively. Asparagine may be synthesized by the action of aspartyl-tRNA amidotransferase [68, 87]. The genome encodes ammonium transporter AmtB and at least one transporter (AroP) for amino acids; thus, ammonium and amino acids are likely the main nitrogen sources. Although the peptidoglycan synthesis pathway remains intact, the pathway for lipopolysaccharide (LPS) synthesis is completely absent.

### Functional reduction and specialization in bacterial symbionts

The CDSs of *P. mixotrichae* and *S. mixotrichae* were classified into COG functional categories for comparisons with their relatives, respectively (Figs. S16 and S17). In *P. mixotrichae*, the number of genes was reduced in many categories only to around half of those of the free-swimming relatives with a similar genome size, including *Leadbettera azotonutricia* and [*Treponema*] *primitia* isolated from termite guts [88–90] (Figs. S9A and S16A; see Supplementary Results). When compared in ratio, a reduction in categories (G) “carbohydrate transport and metabolism” and (T) “signal transduction mechanisms” was conspicuous, whereas increases in categories (J) “translation, ribosomal structure and biogenesis”, (L) “replication, recombination and repair”, and (X) “mobilome: prophages, transposons” were prominent (Fig. S16B). The distribution pattern of COGs in *S. mixotrichae* was largely similar to those of other ectosymbionts in ratio and together exhibited a common reduction in category (T), compared to their free-living relatives (Fig. S17B).

**Figure 4:**
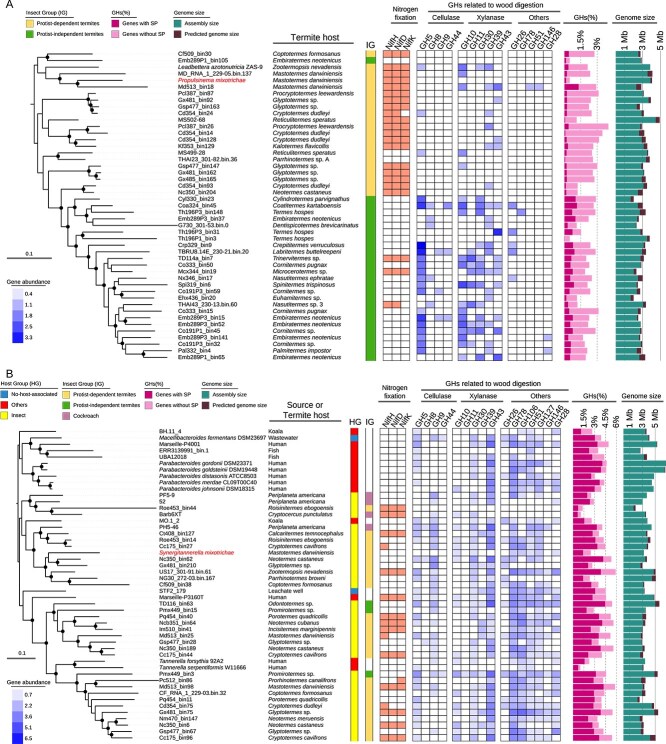
Phylogenomic positions and functions of *Propulsinema mixotrichae* (A) and *Synergitannerella mixotrichae* (B) in comparison with their relatives. (A) A maximum-likelihood (ML) tree was constructed using the LG + F + R6 model based on 5827 aligned amino acid positions. Information concerning reference genomes is shown in Table S4. (B) An ML tree was constructed using the LG + F + R7 model based on 15 879 aligned amino acid positions. Information concerning reference genomes is shown in Table S5. Boxes for “nitrogen fixation” indicate the presence or absence of the *nifHDK* genes. Heatmap for glycoside hydrolases (GHs) indicates the number of genes after log_2_(x + 1) transformation. Only GH genes containing a signal peptide (SP) sequence were counted. “GH (%)” represents the proportion of GH genes in the total number of protein-coding sequences. Genome size was estimated based on assembly size and completeness calculated using CheckM2. Highly supported nodes (ultrafast bootstrap support ≥95%, SH-aLRT ≥80%, 1000 replicates) are indicated with a closed circle. GH26, β-mannanase; GH78 and 106, α-L-rhamnosidase; GH51, α-L-arabinofuranosidase; GH127 and 146, β-L-arabinofuranosidase; GH28, polygalacturonase (pectinase).

The phylogenomic trees of *P. mixotrichae* and *S. mixotrichae* were constructed, respectively, and the presence or absence of *nifHDK* and the number of genes for GHs with a signal peptide were examined (Fig. 4). *Propulsinema mixotrichae* was phylogenetically placed among species with nitrogen-fixing ability and less equipped with GHs for lignocellulose digestion (Fig. 4A). In *Bacteroidales*, nitrogen-fixing ability is sporadic, and the frequency of GH genes with a signal peptide sequence in *S. mixotrichae* was similar or less in comparison with its relatives (Fig. 4B). The repertoire of lignocellulose digestion-related GH genes of *S. mixotrichae* considerably differed from those of other ectosymbiotic *Bacteroidales* associated with flagellates in the gut of the termite *Reticulitermes speratus* [4] or the cockroach *C. punctulatus* (Fig. S18) [67]. Because detailed comparative genomic analyses of *Endomicrobiellum* have recently been reported [68], we do not repeat the analysis here.

### Metabolic complementarity among *M. paradoxa* and its bacterial symbionts

**Figure 5:**
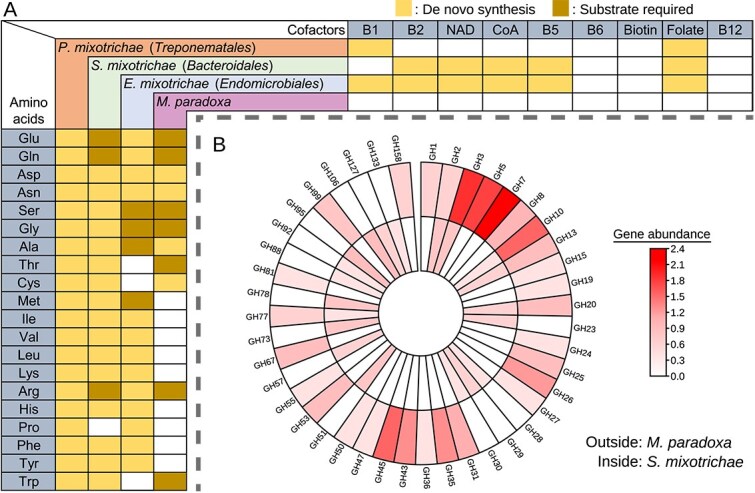
Complementarity between *Mixotricha paradoxa* and its bacterial symbionts. (A) Biosynthetic capacity of amino acids and cofactors. For example, *M. paradoxa* requires 2-oxoglutarate, 5,10-methylene-tetrahydrofolate, L-ornithine, O-phospho-L-homoserine, and indole for the synthesis of glutamine, serine, arginine, threonine, and tryptophan, respectively. The first three of these substrates may be provided by *Endomicrobiellum mixotrichae*. (B) Comparison of glycoside hydrolase (GH) repertoires between *M. paradoxa* and *Synergitannerella mixotrichae*. Heatmap shows the number of genes after log_6_(x + 1) transformation. Redundant homologous GHs in the *M. paradoxa* transcripts were clustered at a 95% similarity threshold. Among the GH families shared by *M. paradoxa* and *S. mixotrichae*, GH2 and GH3 are general oligosaccharide-utilizing enzymes.

Both ectosymbionts can synthesize most of the 20 proteinogenic amino acids. In contrast, *M. paradoxa* and *E. mixotrichae* have limited capacities to synthesize amino acids, which are largely complementary (Fig. 5A). The biosynthetic capability of amino acids is consistent among the *E. mixotrichae* genomes from nine other samples (Tables S3 and S12). In *M. paradoxa* and *S. mixotrichae*, 29 and 20 GH families were identified, respectively, with only six families being shared between the two organisms (Fig. 5B; Table S10). The sole GH family clearly involved in lignocellulose digestion present in both is GH10 (xylanase).

## Discussion

In this study, we predicted the metabolic capacity of the *M. paradoxa* holobiont based on the transcriptome of the flagellate host and the genomes of its dominant ecto- and endosymbionts. Although several studies previously conducted metatranscriptomic or metagenomic analyses of the gut microbiota of protist-dependent termites [91–93], none of these focused on the metabolism of the gut flagellates other than the potential to digest lignocellulose. The hologenome of *Streblomastix strix*, an oxymonad flagellate in the gut of the termite *Zootermopsis angusticollis*, was previously analyzed, and a clear division of metabolic labor was revealed: ectosymbionts belonging to *Bacteroidales* digest lignocellulose, fix dinitrogen, and synthesize nitrogenous compounds, whereas the host *S. strix* provides their habitat but has no capacity to digest lignocellulose and relies almost exclusively on its ectosymbionts for nutrition [14]. However, an equivalent analysis for a parabasalid holobiont has been lacking [69]. Thus, even though many genomic studies have revealed the metabolic capacities of prokaryotic symbionts of parabasalid gut flagellates [10, 12–18, 67], the absence of information on the host’s metabolic capacity has precluded a deeper understanding of the host-symbiont interdependence coevolved in the holobiont.

Our transcriptomic analysis revealed that the core metabolic pathways of *M. paradoxa* (Fig. 2) are almost same with those of *Trichomonas vaginalis* and *Tritrichomonas foetus* [72]*.* The clear difference lies in the repertoire of GH genes, which are characterized by the highly expressed cellulase and hemicellulase genes present in *M. paradoxa* (Fig. S8). These GH genes involved in lignocellulose digestion have possibly been acquired via horizontal gene transfer by an ancestral commensal gut flagellate, although the origins of the genes remain obscure [94].

*Synergitannerella mixotrichae* possesses a diverse array of GH genes, which are largely complementary to the GH repertoire of *M. paradoxa* (Fig. 5B; Table S10). This complementarity implies a synergistic digestion of lignocellulose by this holobiont. The secretory GHs of *S. mixotrichae* include hemicellulases involved in hydrolysis of side chains, and pectinases (Table S10). The lack of cellulase genes in *S. mixotrichae* is rare among *Tannerellaceae*, and the number of secretory GHs is relatively small compared to other *Tannerellaceae* (Fig. 4B). These suggest specialization of the GH repertoire of *S. mixotrichae*. Its GHs might extracellularly “pretreat” wood particles or act together with the host-derived GHs within food vacuoles of *M. paradoxa* (Fig. S2). Further experimental work is required to test these hypotheses.

**Figure 6:**
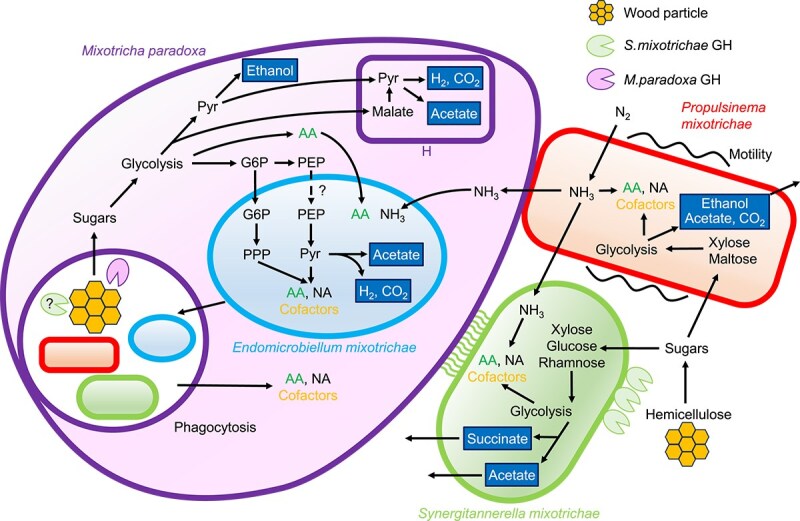
Schematic view of the proposed interaction between *Mixotricha paradoxa* and its bacterial symbionts. Putative metabolic end products (e.g., acetate) are highlighted and framed. Abbreviations: GH, glycosyl hydrolase; G6P, glucose-6-phosphate; PEP, phosphoenolpyruvate; Pyr, pyruvate; PPP, pentose-phosphate pathway; H, hydrogenosomes; AA, amino acids; NA, nucleic acids.

As expected, the genome of *P. mixotrichae* contains an entire set of genes for the assembly of flagella, whereas the expression level of flagellum (cilium)-related genes possessed by *M. paradoxa* was relatively low (Fig. S7). Chemotaxis-related genes are also intact in *P. mixotrichae*, but the trigger for its flagellar movement remains unclear. The nitrogen-fixing ability of *P. mixotrichae* possibly has another critical role in the *M. paradoxa* holobiont, considering the low amount of nitrogen in dead wood ingested by the termite host. Fixed nitrogen may be transferred to *M. paradoxa* and other two bacterial symbionts as ammonia and/or by phagocytosis to *M. paradoxa* (Figs. 6 and S2B–E). The transfer of fixed nitrogen has previously been demonstrated between the parabasalid *Barbulanympha* sp. and its N_2_-fixing ectosymbiont Barb6XT (*Tannerellaceae*) in the gut of *C. punctulatus* (Figs. 4B and S4) [67].

The metabolic interdependence between *M. paradoxa* and its symbionts is most evident in the biosynthetic capability of nitrogenous compounds. *Mixotricha paradoxa* apparently lacks the biosynthetic pathways for production of nucleotides and cofactors (Figs. 2 and 5A), but these can be supplemented possibly by digestion of *E. mixotrichae* and other symbiotic or nonsymbiotic bacteria (Figs. 3 and S2B–E). In addition, *M. paradoxa* and *E. mixotrichae* appear to complement each other’s synthesis of amino acids (Figs. 5A and 6). *Endomicrobiellum mixotrichae* cannot synthesize tryptophan (Figs. 3C and 5A), whereas *M. paradoxa* expressed genes coding for homologs of tryptophan synthase TrpB. No other gene transcripts in the de novo tryptophan biosynthetic pathway were detected in the *M. paradoxa* transcriptomes, and this pattern (i.e. expression of only *trpB*) was previously reported in ciliates [74]. We also found similar expression patterns in previously published transcriptomic data of the pathogenic parabasalid *Histomonas meleagridis* [95] and the cellulolytic parabasalid *P. grassii* [69]. The lack of a tryptophan biosynthetic pathway in many other *Endomicrobiellum* species [68] may be attributable to the expression of TrpB by host parabasalids.

The genome of *E. mixotrichae* exhibited considerable reduction as seen in other *Endomicrobiellum* species [68] and obligate intracellular symbionts in general [25, 96], reflecting its high dependence on the host. In contrast, the genome sizes of the ectosymbionts *P. mixotrichae* and *S. mixotrichae* were similar to their free-living relatives (Fig. S9). Nevertheless, reduction in the number of genes involved in signal transduction is consistent across known ectosymbionts of termite-gut flagellates (Figs. S16 and S17) [4, 18, 67]. This pattern aligns with the hypothesis that the stable environment provided by the flagellate host reduces the need for regulatory mechanisms to adapt to external environmental fluctuations [18].

The absence of enolase is common in the clade containing the MAG “Md513_bin60” (corresponding to *E. mixotrichae*) in *Endomicrobiellum* [68]. We hypothesize that *E. mixotrichae* compensates for this disruption in its glycolytic pathway by using a UhpC/T homolog. It was previously demonstrated that the amino acid properties at positions 388 and 391 of UhpC/T homologs are critical for substrate selectivity [97, 98]. In the G6P transporter UhpT of *Escherichia coli*, when both D388 and K391 sites were replaced by noncharged amino acids such as cysteine, the protein did not lose the ability to import G6P. However, a D388V mutant caused an uncompensated negative charge at K391 and resulted in a substrate shift from G6P to PEP or 3-phosphoglycerate [97, 98]. We thus predict, based on the similarity of amino acids at positions 388 and 391, that MdMp027_0408 imports G6P, MdMp027_0733 imports PEP and exports 2-phosphoglycerate or 3-phosphoglycerate according to the concentration gradient, and MdMp027_0318 imports glycerol-3-phosphate (Fig. S15), which is essential to the biosynthesis of glycerophospholipids in *E. mixotrichae* (Fig. 3C).

Although this study provided fundamental insights into the *M. paradoxa* holobiont, several questions remain unresolved. Because the genome of *M. paradoxa* has not been analyzed and transcripts with low expression levels may have escaped detection, it is not yet possible to fully reveal its metabolic potential. The mechanisms underpinning the role of *P. mixotrichae* as a unique motility symbiont are also not well understood. The coding density of the *P. mixotrichae* genome is lower than most of its relatives (Table S6), the number of its genes in several COG categories was obviously reduced (Fig. S16), and several pseudogenes have been accumulated in category (L) “replication, recombination and repair” (Fig. S19; Supplementary Results). These features may indicate the onset of a significant functional reduction in *P. mixotrichae* and its further integration into this holobiont.

In conclusion, our findings support the view that *M. paradoxa* and its bacterial symbionts form a tightly integrated holobiont, with each partner contributing specialized metabolic functions, i.e. an efficient division of labor. *Mixotricha paradoxa* offers a habitat for its symbionts and digests cellulose and hemicellulose; *P. mixotrichae* provides motility and nitrogen sources; *S. mixotrichae* participates in hemicellulose digestion with GHs complementary to those of *M. paradoxa*; and *E. mixotrichae* exchanges essential compounds with *M. paradoxa* to compensate for deficiencies in biosynthetic capabilities that each partner possesses (Fig. 6).

We propose a plausible coevolutionary scenario that formed this tightly integrated *M. paradoxa* holobiont. Outsourcing motility to ectosymbiotic spirochetes has considerably reduced locomotion costs of the flagellate host and facilitated the evolution of such an unusually large cell capable of ingesting larger wood fragments. The specialized GH repertoire of *S. mixotrichae* complementary to that of *M. paradoxa* has enabled the flagellate to more efficiently digest wood fragments phagocytosed by the large cell. Nitrogen fixation by *P. mixotrichae* and the complementary biosynthetic capabilities of intracellular *E. mixotrichae* have ensured a stable supply of nitrogenous compounds to constitute and maintain the large flagellate cell body without destabilizing the gut ecosystem by devouring free-swimming gut bacteria as nitrogen sources. As the division of labor and resulting interdependence progress, the metabolic efficiency and stability of the holobiont as an integrated entity becomes more strengthened.

The *M. paradoxa* holobiont itself obligately resides within the termite gut; i.e. it is nested within the *M. darwiniensis* holobiont. The *M. darwiniensis* holobiont, in turn, forms a “meta-holobiont” [99] with its nestmates. Holobionts within a meta-holobiont can exchange their microbial symbionts, thereby a holobiont can acquire a superior symbiont [99]. Within this framework, an *M. paradoxa* holobiont with more efficient division of labor and more beneficial to the *M. darwiniensis* holobiont may prevail relatively faster within the *M. darwiniensis* meta-holobiont, increasing the latter’s fitness. This cycle may accelerate the evolution of division of labor within the *M. paradoxa* holobiont.

Under the rules of SeqCode [39], we propose *P. mixotrichae* gen. nov., sp. nov. (*Breznakiellaceae*) for phylotype mpsp15, *S. mixotrichae* gen. nov., sp. nov. (*Tannerellaceae*) for phylotype B6, and *E. mixotrichae* sp. nov. (*Endomicrobiaceae*) for phylotype MdMp-027. The full descriptions of these taxa are given in the Supplementary Results.

## Supplementary Material

Supplementary_Figures_wraf178(1)

Suppelementary_Tables_wraf178

Supplementary_Text_wraf178(1)

VIdeo_S1_wraf178

Video_S1_still_image_wraf178

VIdeo_S2_wraf178

Video_S2_still_image_wraf178

## Data Availability

The sequence data obtained in this study have been deposited in DDBJ under BioProject PRJDB19102. Metagenomic and transcriptomic reads were available at BioSample under accession numbers SAMD00834759–62. The genome sequences of *P. mixotrichae*, *S. mixotrichae*, and *E. mixotrichae* are available under the accession numbers BAAIAF010000001–153, BAAIAG010000001–268, and AP043653, respectively.

## References

[1] Lo N, Eggleton P. Chapter 2: Termite phylogenetics and co-cladogenesis with symbionts. In: Bignell D.E., Roisin Y., Lo N. (eds.), Biology of Termites: A Modern Synthesis. Springer, 2011, 27–50.

[2] Brune A . Symbiotic digestion of lignocellulose in termite guts. *Nat Rev Microbiol* 2014;12:168–80. 10.1038/nrmicro3182 24487819

[3] Pramono AK, Kuwahara H, Itoh T. et al. Discovery and complete genome sequence of a bacteriophage from an obligate intracellular symbiont of a cellulolytic protist in the termite gut. *Microbes Environ* 2017;32:112–7. 10.1264/jsme2.ME16175 28321010 PMC5478533

[4] Yuki M, Kuwahara H, Shintani M. et al. Dominant ectosymbiotic bacteria of cellulolytic protists in the termite gut also have the potential to digest lignocellulose. *Environ Microbiol* 2015;17:4942–53. 10.1111/1462-2920.12945 26079531

[5] Salgado JFM, Hervé V, Vera MAG. et al. Unveiling lignocellulolytic potential: a genomic exploration of bacterial lineages within the termite gut. *Microbiome* 2024;12:201. 10.1186/s40168-024-01917-7 39407345 PMC11481507

[6] Arora J, Kinjo Y, Šobotník J. et al. The functional evolution of termite gut microbiota. *Microbiome* 2022;10:78. 10.1186/s40168-022-01258-3 35624491 PMC9137090

[7] Tokuda G, Mikaelyan A, Fukui C. et al. Fiber-associated spirochetes are major agents of hemicellulose degradation in the hindgut of wood-feeding higher termites. *Proc Natl Acad Sci USA* 2018;115:E11996–2004. 10.1073/pnas.1810550115 30504145 PMC6304966

[8] Beránková T, Arora J, Romero Arias J. et al. Termites and subsocial roaches inherited many bacterial-borne carbohydrate-active enzymes (CAZymes) from their common ancestor. *Commun Biol* 2024;7:e1449. 10.1038/s42003-024-07146-w PMC11541852 39506101

[9] Breznak JA, Brill WJ, Mertins JW. et al. Nitrogen fixation in termites. *Nature* 1973;244:577–80. 10.1038/244577a0 4582514

[10] Hongoh Y, Sharma VK, Prakash T. et al. Genome of an endosymbiont coupling N_2_ fixation to cellulolysis within protist cells in termite gut. *Science* 2008;322:1108–9. 10.1126/science.1165578 19008447

[11] Desai MS, Brune A. *Bacteroidales* ectosymbionts of gut flagellates shape the nitrogen-fixing community in dry-wood termites. *ISME J* 2012;6:1302–13. 10.1038/ismej.2011.194 22189498 PMC3379631

[12] Ohkuma M, Noda S, Hattori S. et al. Acetogenesis from H_2_ plus CO_2_ and nitrogen fixation by an endosymbiotic spirochete of a termite-gut cellulolytic protist. *Proc Natl Acad Sci USA* 2015;112:10224–30. 10.1073/pnas.1423979112 25979941 PMC4547241

[13] Ikeda-Ohtsubo W, Strassert JFH, Köhler T. et al. “*Candidatus* Adiutrix intracellularis”, an endosymbiont of termite gut flagellates, is the first representative of a deep-branching clade of *Deltaproteobacteria* and a putative homoacetogen. *Environ Microbiol* 2016;18:2548–64. 10.1111/1462-2920.13234 26914459

[14] Treitli SC, Kolisko M, Husník F. et al. Revealing the metabolic capacity of *Streblomastix strix* and its bacterial symbionts using single-cell metagenomics. *Proc Natl Acad Sci USA* 2019;116:19675–84. 10.1073/pnas.1910793116 31492817 PMC6765251

[15] Hongoh Y, Sharma VK, Prakash T. et al. Complete genome of the uncultured termite group 1 bacteria in a single host protist cell. *Proc Natl Acad Sci USA* 2008;105:5555–60. 10.1073/pnas.0801389105 18391199 PMC2291132

[16] Strassert JFH, Mikaelyan A, Woyke T. et al. Genome analysis of ‘*Candidatus* Ancillula trichonymphae’, first representative of a deep-branching clade of *Bifidobacteriales*, strengthens evidence for convergent evolution in flagellate endosymbionts. *Environ Microbiol Rep* 2016;8:865–73. 10.1111/1758-2229.12451 27518440

[17] Kuwahara H, Yuki M, Izawa K. et al. Genome of “*Ca*. Desulfovibrio trichonymphae”, an H_2_-oxidizing bacterium in a tripartite symbiotic system within a protist cell in the termite gut. *ISME J* 2017;11:766–76. 10.1038/ismej.2016.143 27801909 PMC5322295

[18] Takeuchi M, Kuwahara H, Murakami T. et al. Parallel reductive genome evolution in *Desulfovibrio* ectosymbionts independently acquired by *Trichonympha* protists in the termite gut. *ISME J* 2020;14:2288–301. 10.1038/s41396-020-0688-1 32483307 PMC7608387

[19] Kaneko M, Omori T, Igai K. et al. Facultative endosymbiosis between cellulolytic protists and methanogenic archaea in the gut of the Formosan termite *Coptotermes formosanus*. *ISME Commun* 2024;4:ycae097. 10.1093/ismeco/ycae097 39081362 PMC11287868

[20] Breznak JA, Switzer JM. Acetate synthesis from H_2_ plus CO_2_ by termite gut microbes. *Appl Environ Microbiol* 1986;52:623–30. 10.1128/aem.52.4.623-630.1986 16347157 PMC239087

[21] Leadbetter JR, Schmidt TM, Graber JR. et al. Acetogenesis from H_2_ plus CO_2_ by spirochetes from termite guts. *Science* 1999;283:686–9. 10.1126/science.283.5402.686 9924028

[22] Noda S, Ohkuma M, Yamada A. et al. Phylogenetic position and *in situ* identification of ectosymbiotic spirochetes on protists in the termite gut. *Appl Environ Microbiol* 2003;69:625–33. 10.1128/AEM.69.1.625-633.2003 12514050 PMC152436

[23] Izawa K, Kuwahara H, Sugaya K. et al. Discovery of ectosymbiotic *Endomicrobium* lineages associated with protists in the gut of stolotermitid termites. *Environ Microbiol Rep* 2017;9:411–8. 10.1111/1758-2229.12549 28556617

[24] Takahashi K, Kuwahara H, Horikawa Y. et al. Emergence of putative energy parasites within *Clostridia* revealed by genome analysis of a novel endosymbiotic clade. *ISME J* 2023;17:1895–906. 10.1038/s41396-023-01502-0 37653056 PMC10579323

[25] Silva JK, Hervé V, Mies US. et al. A novel lineage of endosymbiotic *Actinomycetales*: genome reduction and acquisition of new functions in *Bifidobacteriaceae* associated with termite gut flagellates. *Environ Microbiol* 2025;27:e70010. 10.1111/1462-2920.70010 39778056 PMC11707648

[26] Sato T, Kuwahara H, Fujita K. et al. Intranuclear verrucomicrobial symbionts and evidence of lateral gene transfer to the host protist in the termite gut. *ISME J* 2014;8:1008–19. 10.1038/ismej.2013.222 24335826 PMC3996698

[27] Brugerolle G . Devescovinid features, a remarkable surface cytoskeleton, and epibiotic bacteria revisited in *Mixotricha paradoxa*, a parabasalid flagellate. *Protoplasma.* 2004;224:49–59. 10.1007/s00709-004-0052-8 15726809

[28] Sutherland JL . Protozoa from Australian termites. *Quart J Microscop Sci* 1933;S2-76:145–73.

[29] Yamin MA . Flagellates of the orders Trichomonadida Kirby, Oxymonadida Grasse, and Hypermastigida Grassi & Foa reported from lower termites (Isoptera families Mastotermitidae, Kalotermitidae, Hodotermitidae, Termopsidae, Rhinotermitidae, and Serritermitidae) and from the wood-feeding roach *Cryptocercus* (Dictyoptera: Cryptocercidae). *Sociobiology* 1979;4:5–119.

[30] Cleveland LR, Grimstone AV. The fine structure of the flagellate *Mixotricha paradoxa* and its associated micro-organisms. *Proc R Soc Lond B* 1964;159:668–86. 10.1098/rspb.1964.0025

[31] Cleveland LR, Cleveland BT. The locomotory waves of *Koruga*. *Deltotrichonympha and Mixotricha Archiv Protistenk* 1966;109:39–63.

[32] Wenzel M, Radek R, Brugerolle G. et al. Identification of the ectosymbiotic bacteria of *Mixotricha paradoxa* involved in movement symbiosis. *Eur J Protistol* 2003;39:11–23. 10.1078/0932-4739-00893

[33] Radek R, Nitsch G. Ectobiotic spirochetes of flagellates from the termite *Mastotermes darwiniensis*: attachment and cyst formation. *Eur J Protistol* 2007;43:281–94. 10.1016/j.ejop.2007.06.004 17764914

[34] Berchtold M, König H. Phylogenetic analysis and *in situ* identification of uncultivated spirochetes from the hindgut of the termite *Mastotermes darwiniensis*. *Syst Appl Microbiol* 1996;19:66–73. 10.1016/S0723-2020(96)80011-7

[35] Izawa K, Kuwahara H, Kihara K. et al. Comparison of intracellular “*Ca*. Endomicrobium trichonymphae” genomovars illuminates the requirement and decay of defense systems against foreign DNA. *Genome Biol Evol* 2016;8:3099–107. 10.1093/gbe/evw227 27635050 PMC5174739

[36] Bordenstein SR, Theis KR. Host biology in light of the microbiome: ten principles of holobionts and hologenomes. *PLoS Biol* 2015;13:e1002226. 10.1371/journal.pbio.1002226 26284777 PMC4540581

[37] Wu D, Daugherty SC, van Aken SE. et al. Metabolic complementarity and genomics of the dual bacterial symbiosis of sharpshooters. *PLoS Biol* 2006;4:e188. 10.1371/journal.pbio.0040188 16729848 PMC1472245

[38] Davy SK, Allemand D, Weis VM. Cell biology of cnidarian-dinoflagellate symbiosis. *Microbiol Mol Biol Rev* 2012;76:229–61. 10.1128/MMBR.05014-11 22688813 PMC3372257

[39] Hedlund BP, Chuvochina M, Hugenholtz P. et al. SeqCode: a nomenclatural code for prokaryotes described from sequence data. *Nat Microbiol* 2022;7:1702–8. 10.1038/s41564-022-01214-9 36123442 PMC9519449

[40] Trager W . The cultivation of a cellulose-digesting flagellate, *trichomonas termopsidis*, and of certain other termite protozoa. *Biol Bull* 1934;66:182–90. 10.2307/1537331

[41] Nguyen LT, Schmidt HA, von Haeseler A. et al. IQ-TREE: a fast and effective stochastic algorithm for estimating maximum-likelihood phylogenies. *Mol Biol Evol* 2015;32:268–74. 10.1093/molbev/msu300 25371430 PMC4271533

[42] Ludwig W, Strunk O, Westram R. et al. ARB: a software environment for sequence data. *Nucleic Acids Res* 2004;32:1363–71. 10.1093/nar/gkh293 14985472 PMC390282

[43] Utami YD, Kuwahara H, Murakami T. et al. Phylogenetic diversity and single-cell genome analysis of “*Melainabacteria*”, a non-photosynthetic cyanobacterial group, in the termite gut. *Microbes Environ* 2018;33:50–7. 10.1264/jsme2.ME17137 29415909 PMC5877343

[44] Parks DH, Imelfort M, Skennerton CT. et al. CheckM: assessing the quality of microbial genomes recovered from isolates, single cells, and metagenomes. *Genome Res* 2015;25:1043–55. 10.1101/gr.186072.114 25977477 PMC4484387

[45] Bankevich A, Nurk S, Antipov D. et al. SPAdes: a new genome assembly algorithm and its applications to single-cell sequencing. *J Comput Biol* 2012;19:455–77. 10.1089/cmb.2012.0021 22506599 PMC3342519

[46] Lin HH, Liao YC. Accurate binning of metagenomic contigs via automated clustering sequences using information of genomic signatures and marker genes. *Sci Rep* 2016;6:e24175. 10.1038/srep24175 PMC4828714 27067514

[47] Luo J, Lyu M, Chen R. et al. SLR: a scaffolding algorithm based on long reads and contig classification. *BMC Bioinformatics* 2019;20:e539. 10.1186/s12859-019-3114-9 PMC6820941 31666010

[48] Kolmogorov M, Yuan J, Lin Y. et al. Assembly of long, error-prone reads using repeat graphs. *Nat Biotechnol* 2019;37:540–6. 10.1038/s41587-019-0072-8 30936562

[49] Tanizawa Y, Fujisawa T, Nakamura Y. DFAST: a flexible prokaryotic genome annotation pipeline for faster genome publication. *Bioinformatics* 2018;34:1037–9. 10.1093/bioinformatics/btx713 29106469 PMC5860143

[50] Aziz RK, Bartels D, Best A. et al. The RAST server: rapid annotations using subsystems technology. *BMC Genomics* 2008;9:e75. 10.1186/1471-2164-9-75 PMC2265698 18261238

[51] Altschul SF, Gish W, Miller W. et al. Basic local alignment search tool. *J Mol Biol* 1990;215:403–10. 10.1016/S0022-2836(05)80360-2 2231712

[52] Moriya Y, Itoh M, Okuda S. et al. KAAS: an automatic genome annotation and pathway reconstruction server. *Nucleic Acids Res* 2007;35:W182–5. 10.1093/nar/gkm321 17526522 PMC1933193

[53] Kanehisa M, Sato Y. KEGG mapper for inferring cellular functions from protein sequences. *Protein Sci* 2020;29:28–35. 10.1002/pro.3711 31423653 PMC6933857

[54] Zheng J, Ge Q, Yan Y. et al. DbCAN3: automated carbohydrate-active enzyme and substrate annotation. *Nucleic Acids Res* 2023;51:W115–21. 10.1093/nar/gkad328 37125649 PMC10320055

[55] Abby SS, Cury J, Guglielmini J. et al. Identification of protein secretion systems in bacterial genomes. *Sci Rep* 2016;6:e23080. 10.1038/srep23080 PMC4793230 26979785

[56] Mulkidjanian AY, Galperin MY, Makarova KS. et al. Evolutionary primacy of sodium bioenergetics. *Biol Direct* 2008;3:e13. 10.1186/1745-6150-3-13 PMC2359735 18380897

[57] Marchler-Bauer A, Panchenko AR, Shoemarker BA. et al. CDD: a database of conserved domain alignments with links to domain three-dimensional structure. *Nucleic Acids Res* 2002;30:281–3. 10.1093/nar/30.1.281 11752315 PMC99109

[58] Parks DH, Chuvochina M, Chaumeil PA. et al. A complete domain-to-species taxonomy for bacteria and archaea. *Nat Biotechnol* 2020;38:1079–86. 10.1038/s41587-020-0501-8 32341564

[59] Chklovski A, Parks DH, Woodcroft BJ. et al. CheckM2: a rapid, scalable and accurate tool for assessing microbial genome quality using machine learning. *Nat Methods* 2023;20:1203–12. 10.1038/s41592-023-01940-w 37500759

[60] Jain C, Rodriguez-R LM, Phillippy AM. et al. High throughput ANI analysis of 90K prokaryotic genomes reveals clear species boundaries. *Nat Commun* 2018;9:e5114. 10.1038/s41467-018-07641-9 PMC6269478 30504855

[61] Grabherr MG, Haas BJ, Yassour M. et al. Full-length transcriptome assembly from RNA-Seq data without a reference genome. *Nat Biotechnol* 2011;29:644–52. 10.1038/nbt.1883 21572440 PMC3571712

[62] Simão FA, Waterhouse RM, Ioannidis P. et al. BUSCO: assessing genome assembly and annotation completeness with single-copy orthologs. *Bioinformatics* 2015;31:e323. 10.1093/bioinformatics/btv351 26059717

[63] Patro R, Duggal G, Love MI. et al. Salmon provides fast and bias-aware quantification of transcript expression. *Nat Methods* 2017;14:417–9. 10.1038/nmeth.4197 28263959 PMC5600148

[64] Brune A, Song Y, Oren A. et al. A new family for ‘termite gut treponemes’: description of *Breznakiellaceae* fam. nov., *Gracilinema caldarium* gen. nov., comb. nov., *Leadbettera azotonutricia* gen. nov., comb. nov., *Helmutkoenigia isoptericolens* gen. nov., comb. nov., and *Zuelzera stenostrepta* gen. nov., comb. nov., and proposal of *Rectinemataceae* fam. nov. *Int J Syst Evol Microbiol* 2022;72:e005439. 10.1099/ijsem.0.005439 35639582

[65] Stingl U, Maass A, Radek R. et al. Symbionts of the gut flagellate *Staurojoenina* sp. from *Neotermes cubanus* represent a novel, termite-associated lineage of *Bacteroidales*: description of “*Candidatus* Vestibaculum illigatum”. *Microbiology* 2004;150:2229–35. 10.1099/mic.0.27135-0 15256565

[66] Strassert JFH, Desai MS, Radek R. et al. Identification and localization of the multiple bacterial symbionts of the termite gut flagellate *Joenia annectens*. *Microbiology* 2010;156:2068–79. 10.1099/mic.0.037267-0 20378649

[67] Tai V, Carpenter KJ, Weber PK. et al. Genome evolution and nitrogen fixation in bacterial ectosymbionts of a protist inhabiting wood-feeding cockroaches. *Appl Environ Microbiol* 2016;82:4682–95. 10.1128/AEM.00611-16 27235430 PMC4984305

[68] Mies US, Hervé V, Kropp T. et al. Genome reduction and horizontal gene transfer in the evolution of *Endomicrobia*—rise and fall of an intracellular symbiosis with termite gut flagellates. *mBio* 2024;15:e00826–4. 10.1128/mbio.00826-24 38742878 PMC11257099

[69] Nishimura Y, Otagiri M, Yuki M. et al. Division of functional roles for termite gut protists revealed by single-cell transcriptomes. *ISME J* 2020;14:2449–60. 10.1038/s41396-020-0698-z 32514117 PMC7490689

[70] Abdel-Glil MY, Solle J, Wibberg D. et al. Chromosome-level genome assembly of *Tritrichomonas foetus*, the causative agent of bovine Trichomonosis. *Sci Data* 2024;11:e1030. 10.1038/s41597-024-03818-8 PMC11415386 39304666

[71] Carlton JM, Hirt RP, Silva JC. et al. Draft genome sequence of the sexually transmitted pathogen *Trichomonas vaginalis*. *Science* 2007;315:207–12. 10.1126/science.1132894 17218520 PMC2080659

[72] Müller M, Mentel M, van Hellemond JJ. et al. Biochemistry and evolution of anaerobic energy metabolism in eukaryotes. *Microbiol Mol Biol Rev* 2012;76:444–95. 10.1128/MMBR.05024-11 22688819 PMC3372258

[73] Smutná T, Dohnálková A, Sutak R. et al. A cytosolic ferredoxin-independent hydrogenase possibly mediates hydrogen uptake in *Trichomonas vaginalis*. *Curr Biol* 2022;32:124–135.e5. 10.1016/j.cub.2021.10.050 34762819

[74] Imanian B, Keeling PJ. Horizontal gene transfer and redundancy of tryptophan biosynthetic enzymes in dinotoms. *Genome Biol Evol* 2014;6:333–43. 10.1093/gbe/evu014 24448981 PMC3942023

[75] Raymond J, Siefert JL, Staples CR. et al. The natural history of nitrogen fixation. *Mol Biol Evol* 2004;21:541–54. 10.1093/molbev/msh047 14694078

[76] Kobe B, Deisenhofer J. The leucine-rich repeat: a versatile binding motif. *Trends Biochem Sci* 1994;19:415–21. 10.1016/0968-0004(94)90090-6 7817399

[77] Konkel ME, Larson CL, Flanagan RC. *Campylobacter jejuni* FlpA binds fibronectin and is required for maximal host cell adherence. *J Bacteriol* 2010;192:68–76. 10.1128/JB.00969-09 19880595 PMC2798237

[78] Jernigan KK, Bordenstein SR. Ankyrin domains across the tree of life. *PeerJ* 2014;2:e264. 10.7717/peerj.264 24688847 PMC3932732

[79] Martens EC, Koropatkin NM, Smith TJ. et al. Complex glycan catabolism by the human gut microbiota: the Bacteroidetes Sus-like paradigm. *J Biol Chem* 2009;284:24673–7. 10.1074/jbc.R109.022848 19553672 PMC2757170

[80] McBride MJ, Nakane D. *Flavobacterium* gliding motility and the type IX secretion system. *Curr Opin Microbiol* 2015;28:72–7. 10.1016/j.mib.2015.07.016 26461123

[81] Veith PD, Nor Muhammad NA, Dashper SG. et al. Protein substrates of a novel secretion system are numerous in the Bacteroidetes phylum and have in common a cleavable C-terminal secretion signal, extensive post-translational modification, and cell-surface attachment. *J Proteome Res* 2013;12:4449–61. 10.1021/pr400487b 24007199

[82] Nelson SS, Bollampalli S, McBride MJ. SprB is a cell surface component of the *Flavobacterium johnsoniae* gliding motility machinery. *J Bacteriol* 2008;190:2851–7. 10.1128/JB.01904-07 18281397 PMC2293251

[83] Shrivastava A, Rhodes RG, Pochiraju S. et al. *Flavobacterium johnsoniae* RemA is a mobile cell surface lectin involved in gliding. *J Bacteriol* 2012;194:3678–88. 10.1128/JB.00588-12 22582276 PMC3393505

[84] Narita Y, Sato K, Yukitake H. et al. Lack of a surface layer in *Tannerella forsythia* mutants deficient in the type IX secretion system. *Microbiology* 2014;160:2295–303. 10.1099/mic.0.080192-0 25023245 PMC4175972

[85] Kharade SS, McBride MJ. *Flavobacterium johnsoniae* chitinase ChiA is required for chitin utilization and is secreted by the type IX secretion system. *J Bacteriol* 2014;196:961–70. 10.1128/JB.01170-13 24363341 PMC3957688

[86] Zhu Y, McBride MJ. The unusual cellulose utilization system of the aerobic soil bacterium *Cytophaga hutchinsonii*. *Appl Microbiol Biotechnol* 2017;101:7113–27. 10.1007/s00253-017-8467-2 28849247

[87] Min B, Pelaschier JT, Graham DE. et al. Transfer RNA-dependent amino acid biosynthesis: an essential route to asparagine formation. *Proc Natl Acad Sci USA* 2002;99:2678–83. 10.1073/pnas.012027399 11880622 PMC122407

[88] Graber JR, Leadbetter JR, Breznak JA. Description of *Treponema azotonutricium* sp. nov. and *Treponema primitia* sp. nov., the first spirochetes isolated from termite guts. *Appl Environ Microbiol* 2004;70:1315–20. 10.1128/AEM.70.3.1315-1320.2004 15006748 PMC368361

[89] Rosenthal AZ, Matson EG, Eldar A. et al. RNA-seq reveals cooperative metabolic interactions between two termite-gut spirochete species in co-culture. *ISME J* 2011;5:1133–42. 10.1038/ismej.2011.3 21326336 PMC3146290

[90] Ballor NR, Paulsen I, Leadbetter JR. Genomic analysis reveals multiple [FeFe] hydrogenases and hydrogen sensors encoded by treponemes from the H_2_-rich termite gut. *Microb Ecol* 2012;63:282–94. 10.1007/s00248-011-9922-8 21811792

[91] Todaka N, Moriya S, Saita K. et al. Environmental cDNA analysis of the genes involved in lignocellulose digestion in the symbiotic protist community of *Reticulitermes speratus*. *FEMS Microbiol Ecol* 2007;59:592–9. 10.1111/j.1574-6941.2006.00237.x 17239084

[92] Yuki M, Moriya S, Inoue T. et al. Transcriptome analysis of the digestive organs of *Hodotermopsis sjostedti*, a lower termite that hosts mutualistic microorganisms in its hindgut. *Zool Sci* 2008;25:401–6. 10.2108/zsj.25.401 18459822

[93] Tartar A, Wheeler MM, Zhou X. et al. Parallel metatranscriptome analyses of host and symbiont gene expression in the gut of the termite *Reticulitermes flavipes*. *Biotechnol Biofuels* 2009;2:e25. 10.1186/1754-6834-2-25 PMC2768689 19832970

[94] Todaka N, Inoue T, Saita K. et al. Phylogenetic analysis of cellulolytic enzyme genes from representative lineages of termites and a related cockroach. *PLoS One* 2010;5:e8636. 10.1371/journal.pone.0008636 20072608 PMC2797642

[95] Mazumdar R, Endler L, Monoyios A. et al. Establishment of a de novo reference transcriptome of *Histomonas meleagridis* reveals basic insights about biological functions and potential pathogenic mechanisms of the parasite. *Protist* 2017;168:663–85. 10.1016/j.protis.2017.09.004 29107797

[96] McCutcheon JP, Moran NA. Extreme genome reduction in symbiotic bacteria. *Nat Rev Microbiol* 2012;10:13–26. 10.1038/nrmicro2670 22064560

[97] Hall JA, Fann MC, Maloney PC. Altered substrate selectivity in a mutant of an intrahelical salt bridge in UhpT, the sugar phosphate carrier of *Escherichia coli*. *J Biol Chem* 1999;274:6148–53. 10.1074/jbc.274.10.6148 10037698

[98] Hall JA, Maloney PC. Transmembrane segment 11 of UhpT, the sugar phosphate carrier of *Escherichia coli*, is an α-helix that carries determinants of substrate selectivity. *J Biol Chem* 2001;276:25107–13. 10.1074/jbc.M102017200 11349129

[99] Vannier N, Mony C, Bittebiere AK. et al. Clonal plants as meta-holobionts. *mSystems* 2019;4:e00213–8. 10.1128/mSystems.00213-18 30944875 PMC6426648

