# Supplementary material for: Functional division of labor in motility, lignocellulose digestion, and nitrogen metabolism revealed for the Mixotricha paradoxa holobiont
Source: ISME J. 2025 Aug 20;19(1):wraf178. doi: 10.1093/ismejo/wraf178 (PMC12483993; doi:10.1093/ismejo/wraf178)
Supplement: Supplementary_Figures_wraf178(1) [file supplementary_figures_wraf178(1).pdf]

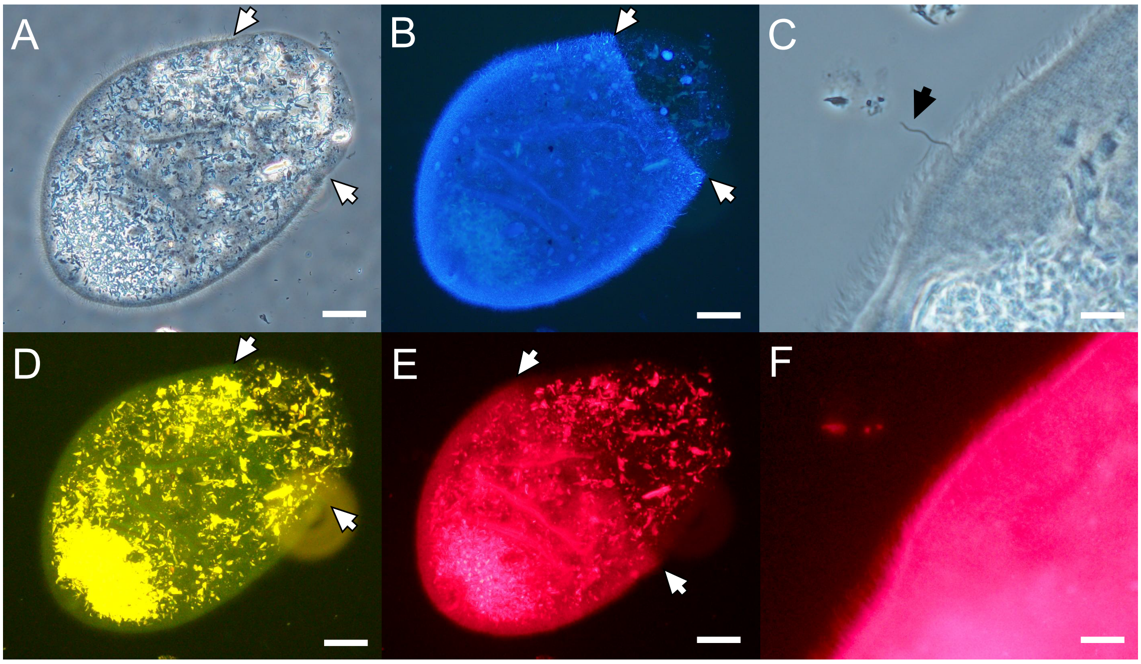

Figure S1. Detection of *Propulsinema mixotrichae* and *Synergitannerella mixotrichae* on the cell surface of *Mixotricha paradoxa* by fluorescence in situ hybridization (FISH). (**A**) Phase-contrast image of *M. paradoxa*. (**B**) DAPI stain (blue). White arrows indicate the boundary of area densely covered by ectosymbionts. The posterior area without ectosymbionts is likely used for phagocytosis of wood particles. (**C**) Magnified phase-contrast image showing short spirochetes covering the host cell surface and a long spirochete indicated by black arrow. (**D–F**) Epifluorescence images. (**D**) Detection of *S. mixotrichae* using probe MdMp-bact197 (6FAM-labeled, green) (Table S1). White arrows indicate the boundary of area densely covered by *S. mixotrichae*. Yellow fragments are wood particles phagocytosed by *M. paradoxa*. (**E**) Detection of *P. mixotrichae* using probe MdMp-014-133 (Texas red-labeled, red) (Table S1). White arrows indicate the boundary of area densely covered by *P. mixotrichae*. (**F**) Magnified view of panel E, corresponding to the region of panel C. Only short spirochetes were detected by the FISH analysis. Bars: (A, B, D, E) 50 µm; (C, F) 10 µm.

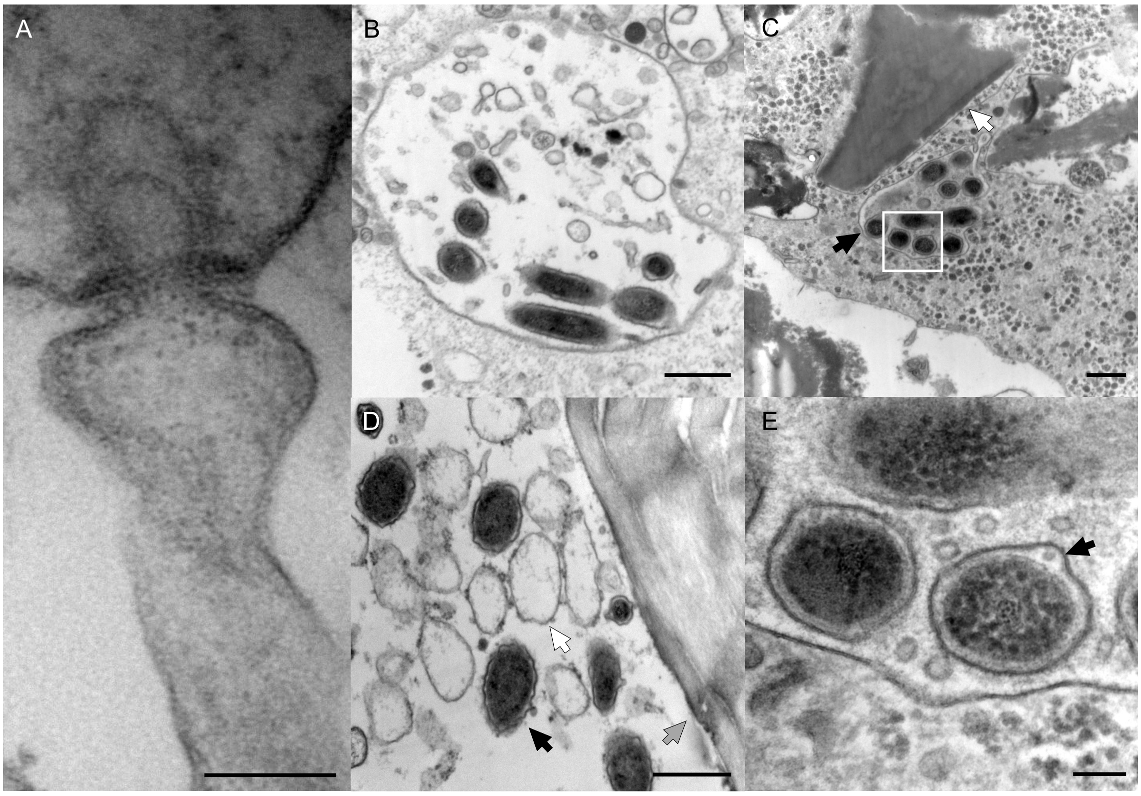

Figure S2. Transmission electron micrographs of *Mixotricha paradoxa* and its symbiotic bacteria. (**A**) Insertion of the anterior end of *Propulsinema mixotrichae* into the invagination of the cell surface of *M. paradoxa*. (**B**) Food vacuole of *M. paradoxa* containing several bacterial cells. (**C**) Food vacuole-like structure containing several bacterial cells (black arrow). White arrow indicates a food vacuole filled with a wood fragment. (**D**) Bacteria resembling *Endomicrobiellum mixotrichae* (black arrow, Fig. 1H) are observed within a food vacuole-like structure, alongside wood fragments (gray arrow) and remnants of digested bacterial cells (white arrow). (**E**) Magnified image of the area indicated in panel C. Endoplasmic flagellum (black arrow), characteristic to spirochetes, is visible in several bacterial cells [45]. Bars: (A, E) 100 nm; (B, C, D) 500 nm.

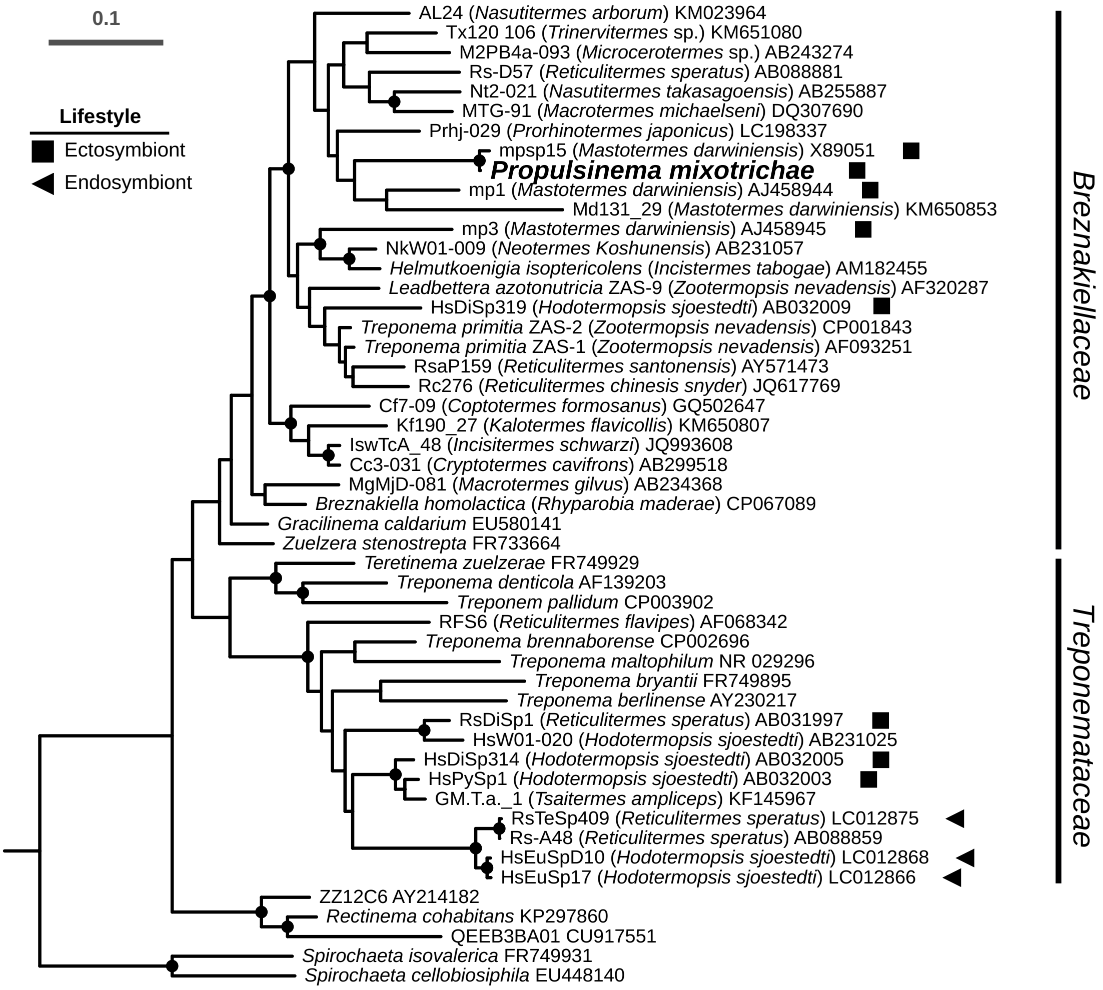

Figure S3. Phylogenetic position of *Propulsinema mixotrichae* based on 16S rRNA gene. Maximum-likelihood tree was constructed using the TIM3+F+R4 model based on 1,462 aligned positions, with *Spirochaeta isovalerica* (FR749931) and *Spirochaeta cellobiosiphila* (EU448140) used as outgroups. Host termite or cockroach species are shown in parentheses. Highly supported nodes (ultrafast bootstrap support ≥ 95%, SH-aLRT ≥ 80%, 1,000 replicates) are indicated with a closed circle. Taxonomy was based on GTDB.

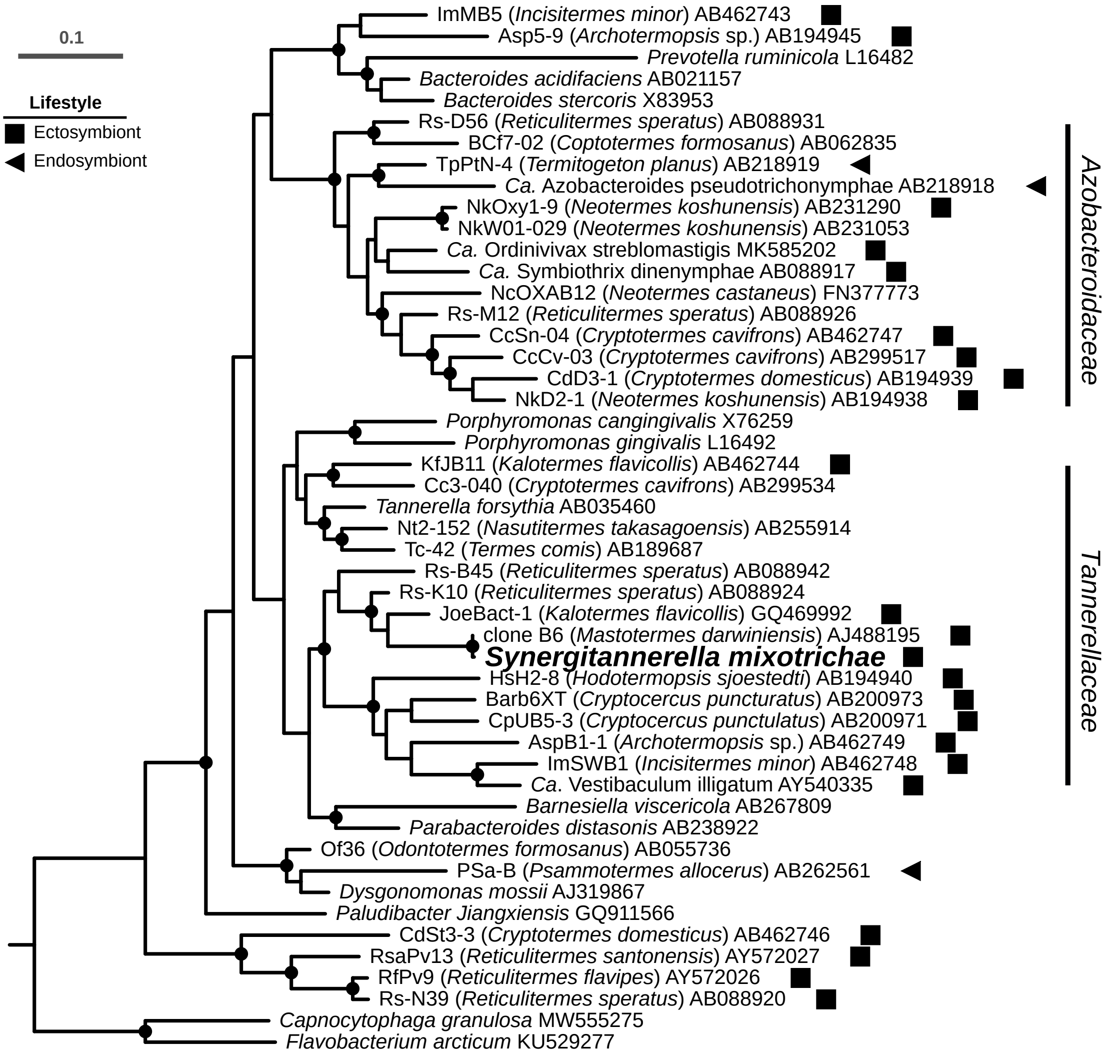

Figure S4. Phylogenetic position of *Synergitannerella mixotrichae* based on 16S rRNA gene. Maximum-likelihood tree was constructed using the GTR+F+R4 model based on 1,352 aligned positions, with *Capnocytophaga granulosa* (MW555275) and *Flavobacterium arcticum* (KU529277) used as outgroups. Host termite or cockroach species are shown in parentheses. Highly supported nodes (ultrafast bootstrap support ≥ 95%, SH-aLRT ≥ 80%, 1,000 replicates) are indicated with a closed circle. Taxonomy was based on GTDB.

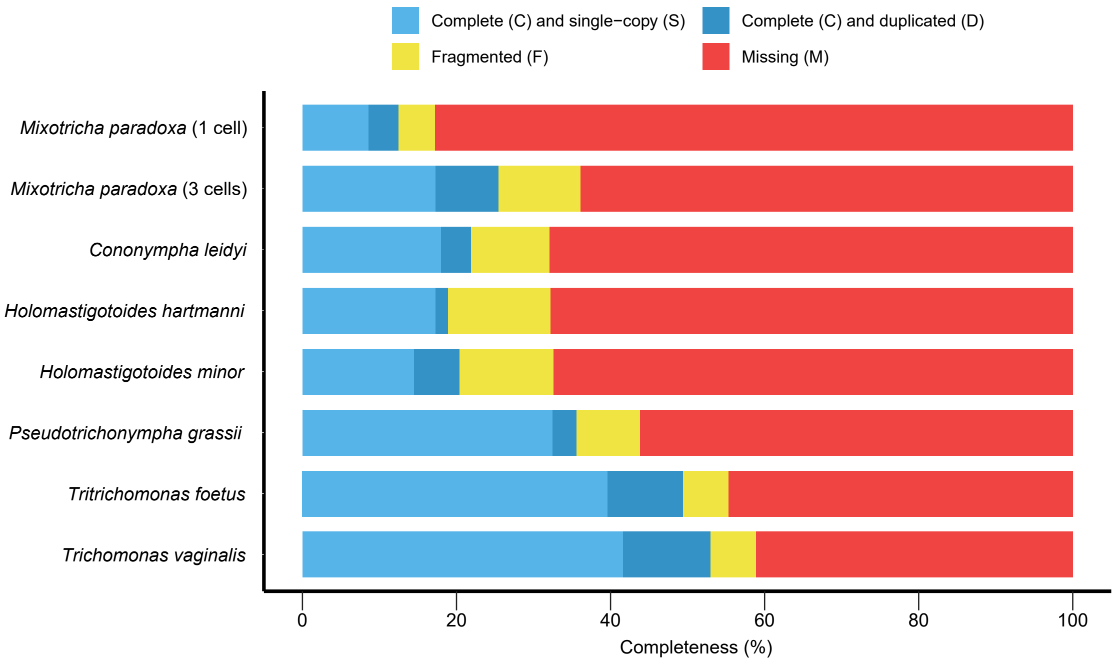

Figure S5. Comparison of transcriptome or genome completeness between *Mixotricha paradoxa* and other parabasalid flagellates. Transcriptomes of four parabasalid flagellates (i.e., *Cononympha leidyi*, *Holomastigotoides hartmanni*, *Holomastigotoides minor*, *Pseudotrichonympha grassii*) in the gut of the termite *Coptotermes formosanus* were obtained from a previous study [46]. The genomes of *Tritrichomonas foetus* and *Trichomonas vaginalis* were retrieved from TrichDB release 57 (https://trichdb.org/trichdb/app), respectively. The completeness was estimated using BUSCO v4.0.6 with the dataset "eukaryota_odb10.2019-11-2".

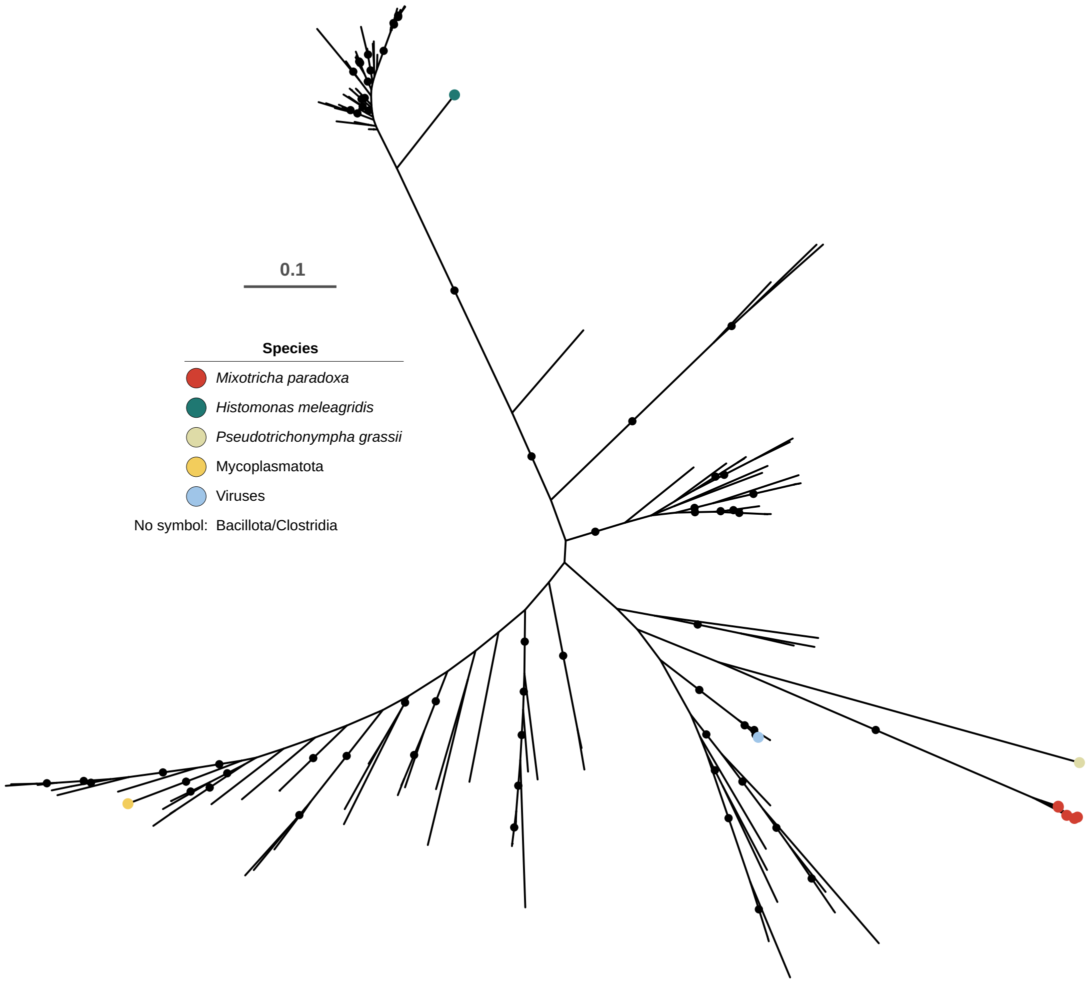

Figure S6. Phylogenetic positions of TrpB expressed by parabasalid flagellates. A maximum-likelihood tree was constructed using the LG+I+G4 model based on 454 aligned positions. Transcripts of *trpB* were detected both in the single-cell and three-cell *M. paradoxa* samples and were phylogenetically closest to TrpB expressed by *Pseudotrichonympha grassii* in the gut of *Coptotermes formosanus* [46]. Highly supported nodes (ultrafast bootstrap support ≥ 95%, SH-aLRT ≥ 80%, 1,000 replicates) are indicated with a closed circle.

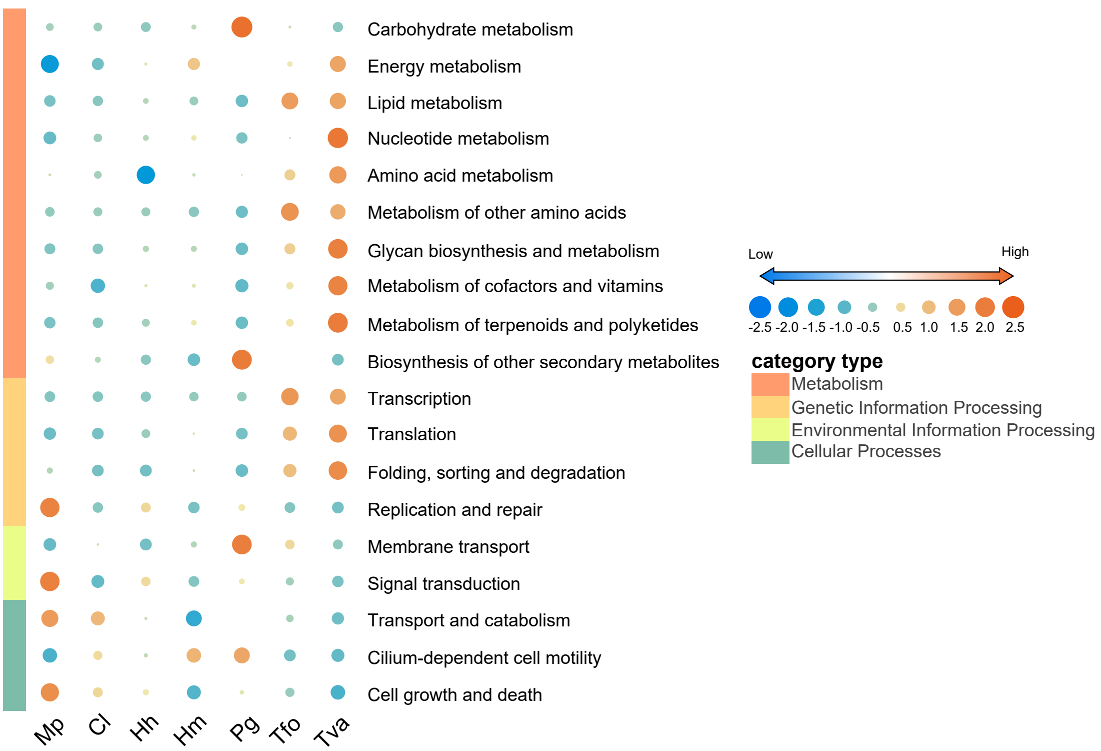

Figure S7. Transcriptome profile of *Mixotricha paradoxa* and other parabasalid flagellates. The functional categories were assigned based on KEGG orthology. Abundance was calculated as transcripts per million and subjected to row normalization. The data for *M. paradoxa* (Mp) are mean values of the single and three-cell samples. The data for other flagellate species are mean values of three samples reported previously. Cl: *Cononympha leidyi*; Hh: *Holomastigotoides hartmanni*; Hm: *Holomastigotoides minor*; Pg: *Pseudotrichonympha grassii* (DRR1852229 to DRR185224) [46]; Tfo: *Tritrichomonas foetus* (ERR4352424, ERR4398931, and ERR4398932); Tva: *Trichomonas vaginalis* (SRR2132589 to SRR2132591).

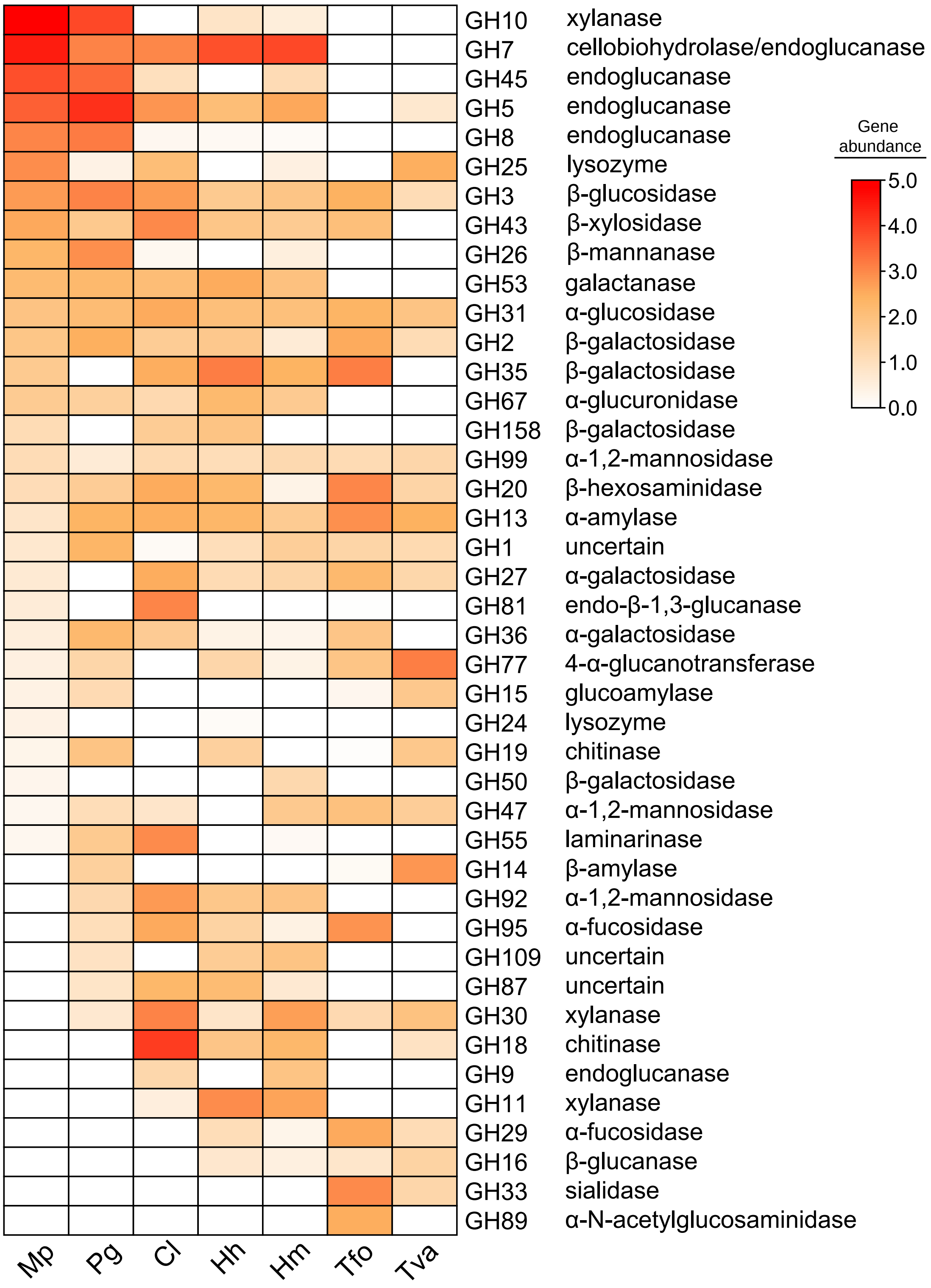

Figure S8. Glycoside hydrolase (GH) genes expressed by *Mixotricha paradoxa* and other parabasalid flagellates. Abundance was calculated as transcripts per million (TPM) and subjected to $\log_{10}(x+1)$ transformation for display in the heatmap. The vertical axis

includes all GH families expressed by *M. paradoxa* as well as the top 10 GH families expressed by the other parabasalid species. The order of GH families is based on TPM in *M. paradoxa* (Mp). The data for *M. paradoxa* are mean values of the single and three-cell samples. The data for other flagellate species are mean values of three samples reported previously. Cl: *Cononympha leidyi*; Hh: *Holomastigotoides hartmanni*; Hm: *Holomastigotoides minor*; Pg: *Pseudotrichonympha grassii* (DRR1852229 to DRR185224) [46]; Tfo: *Tritrichomonas foetus* (ERR4352424, ERR4398931, and ERR4398932); Tva: *Trichomonas vaginalis* (SRR2132589 to SRR2132591).

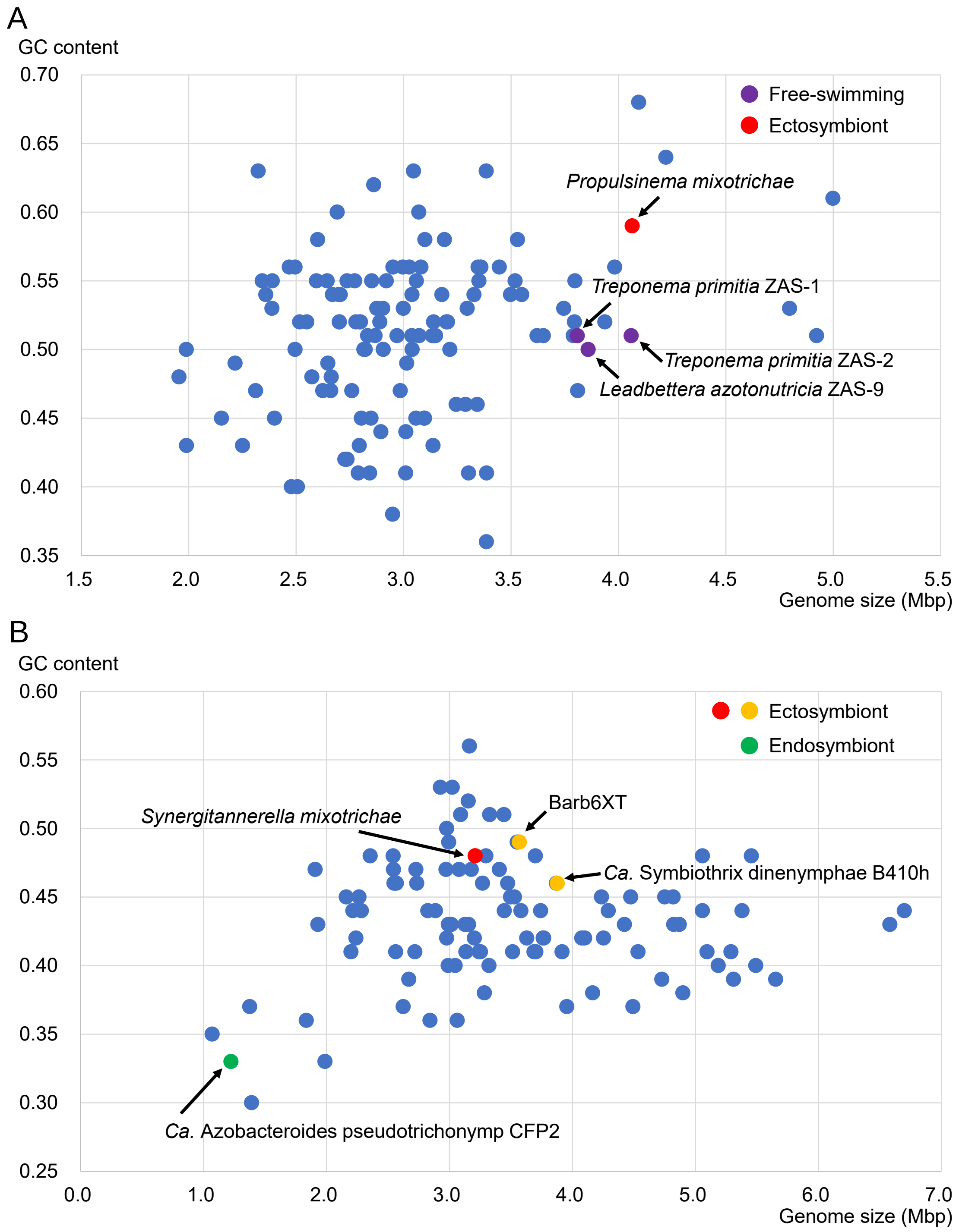

Figure S9. GC content and genome size of *Propulsinema mixotrichae* (A) and *Synergitannerella mixotrichae* (B) in comparison with their respective relatives. The metagenome-assembled genome of "*Candidatus* Ordinivivax streblomastigis", which is an ectosymbiont of the oxymonad flagellate *Streblomastix strix* [47] was not included in panel B due to its low genome completeness (< 80%).

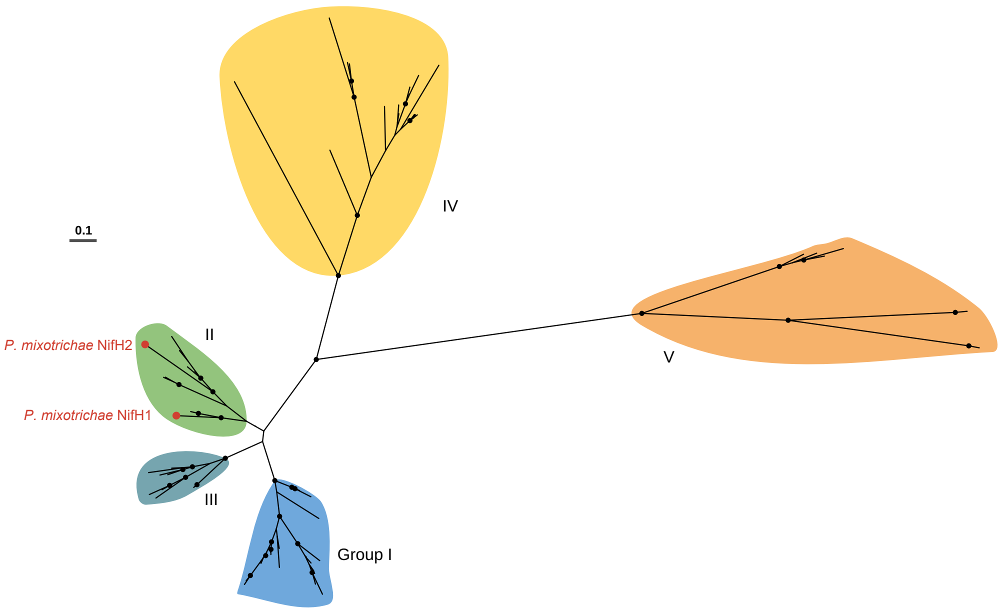

Figure S10. Phylogenetic assignment of two NifH homologs of *Propulsinema mixotrichae* into NifH Groups I–V. A maximum-likelihood tree was constructed using the LG+G4 model based on 221 aligned amino acid positions. Highly supported nodes (ultrafast bootstrap support ≥ 95%, SH-aLRT ≥ 80%, 1,000 replicates) are indicated with a closed circle. The NifH groups have been proposed by a previous study [48].

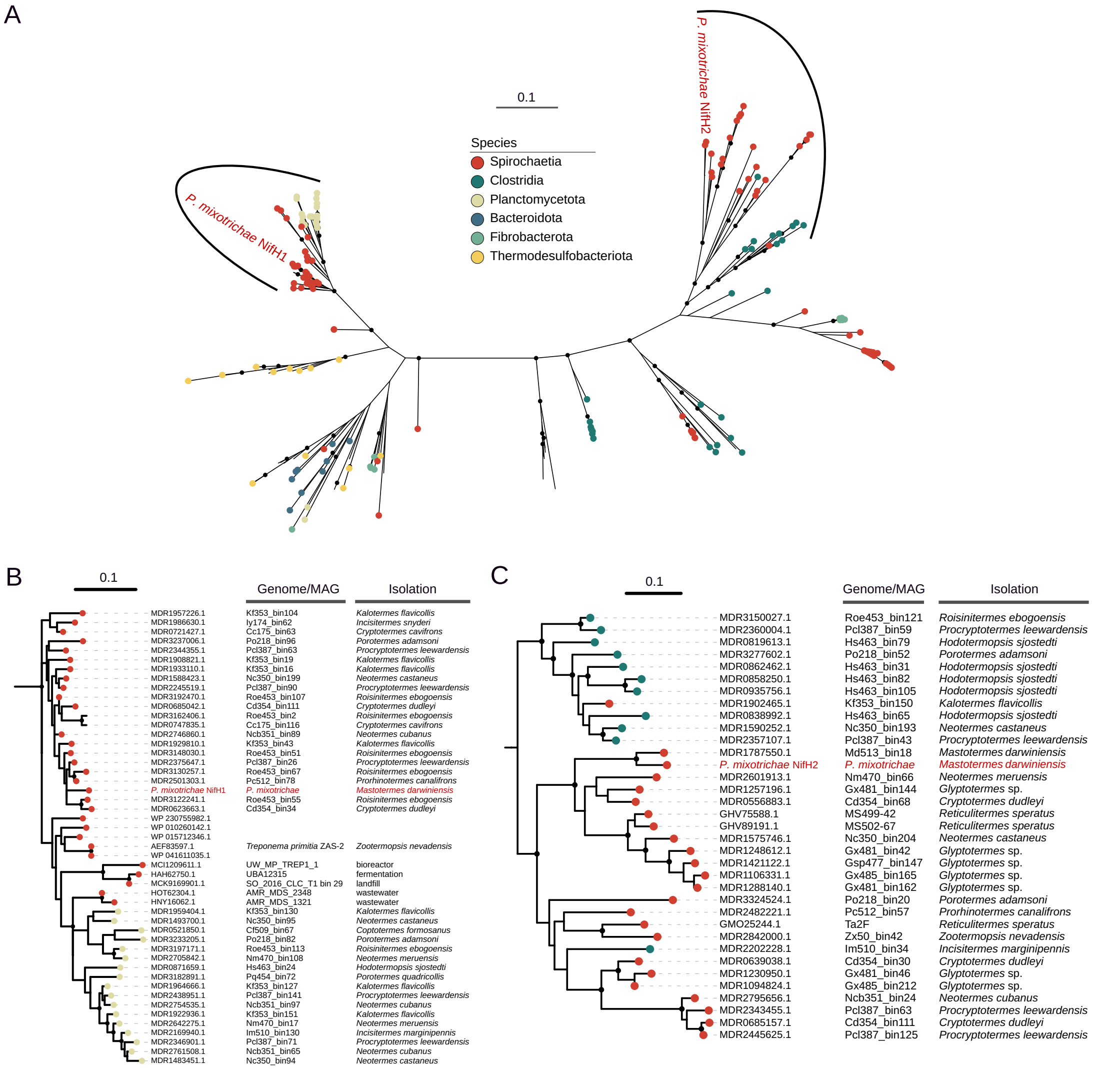

Figure S11. Phylogenetic positions of two NifH homologs of *Propulsinema mixotrichae* within NifH Group II. (**A**) Phylogenetic positions of NifH1 and NifH2 of *P. mixotrichae* among the whole Group II sequences. (**B**) Detailed position of NifH1 of *P. mixotrichae*. (**C**) Detailed position of NifH2 of *P. mixotrichae*. A maximum-likelihood tree was constructed using the LG+R5 model based on 272 aligned amino acid positions. Highly supported nodes (ultrafast bootstrap support ≥ 95%, SH-aLRT ≥ 80%, 1,000 replicates) are indicated with a closed circle.

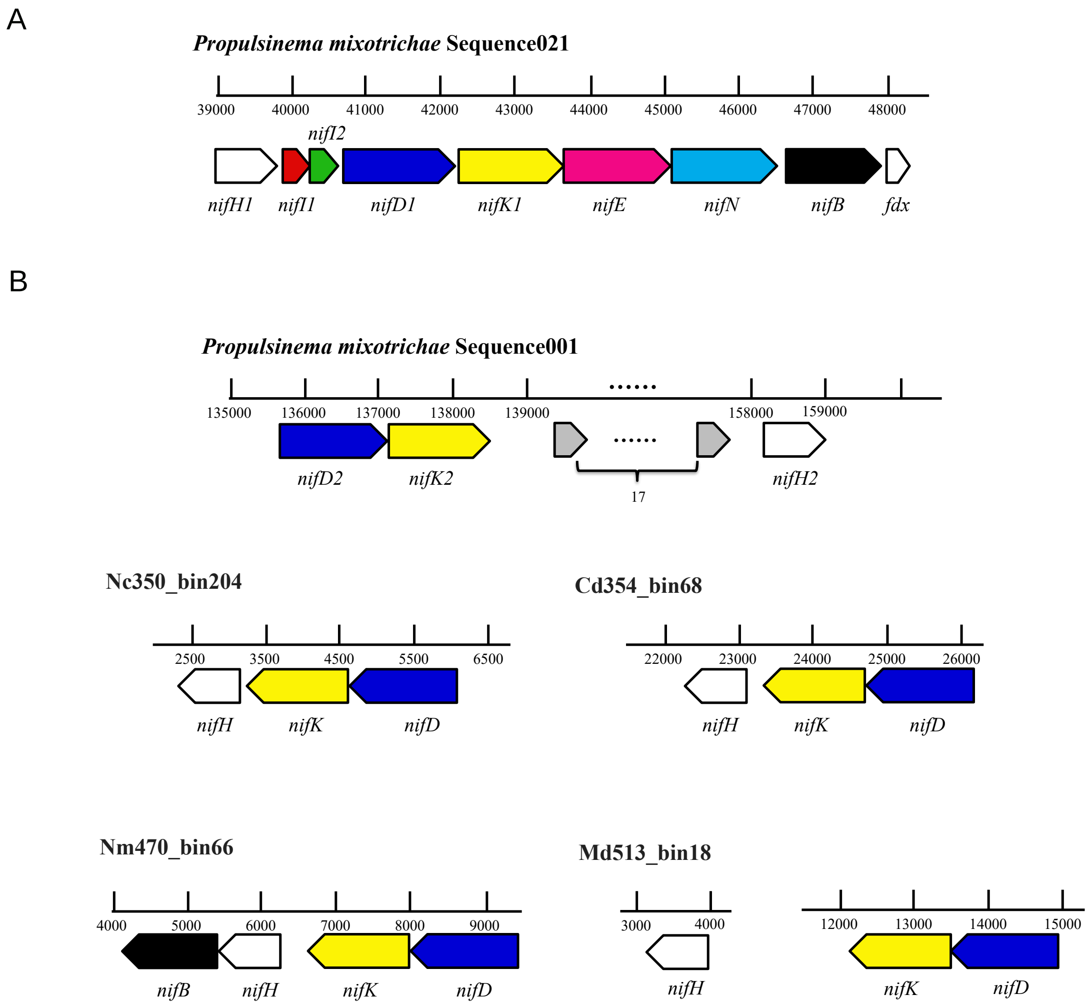

Figure S12. Structures of *nif* gene clusters. (**A**) Gene cluster containing *nifH1* of *Propulsinema mixotrichae*. It forms a typical FeMo-dependent nitrogenase operon with *nifHDKENB* with nitrogen regulatory protein PII genes, *nifI1* and *nifI2*. (**B**) Gene cluster containing *nifH2* of *P. mixotrichae* and clusters with its homologs in reference metagenome-assembled genomes from termite guts. The *nifH2* gene do not form an operon with *nifDK*, and its homologs also do not constitute a typical *nif*-operon for nitrogen fixation.

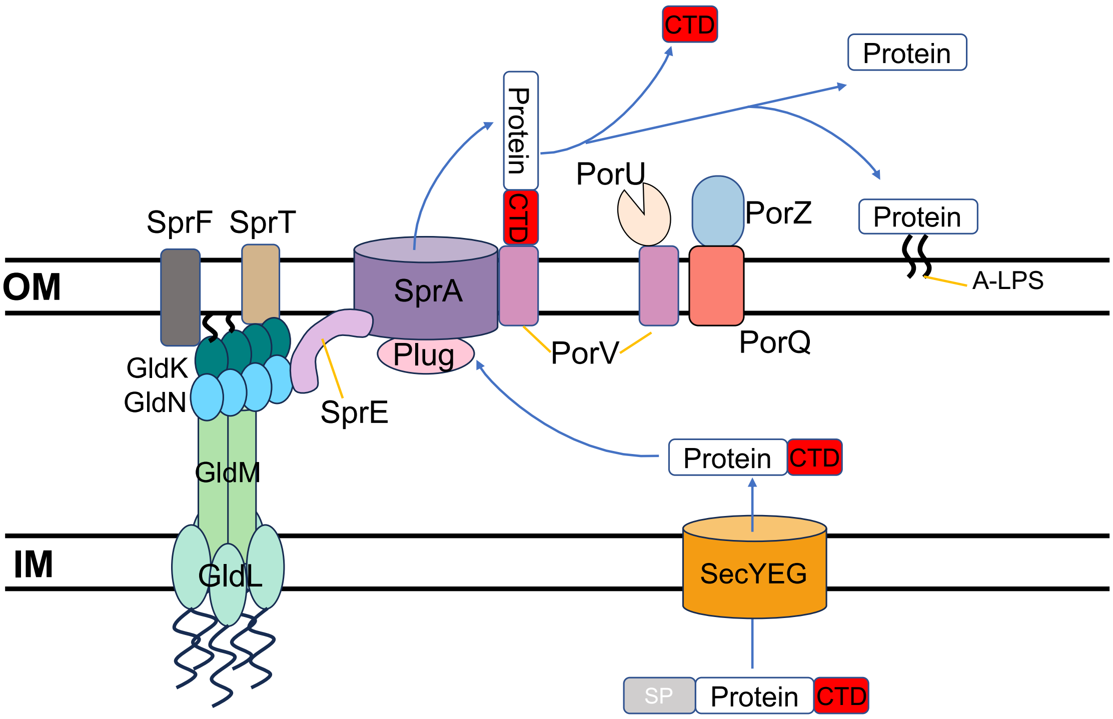

Figure S13. Schematic representation of the type IX secretion system (T9SS) encoded by the genome of *Synergitannerella mixotrichae*. A substrate of T9SS generally contains both a signal peptide (SP) and a conserved C-terminal domain (CTD). The SP enables recognition by the SEC system for transport to the periplasm, after which the CTD is recognized by the T9SS. The substrate is transferred to the PorV shuttle via the SprA translocon and then transported to the PorQUZ complex. Finally, the PorU sortase cleaves the CTD, releasing the protein into the medium or anchoring it to the cell surface. The schematic diagram is based on previous studies [49,50].

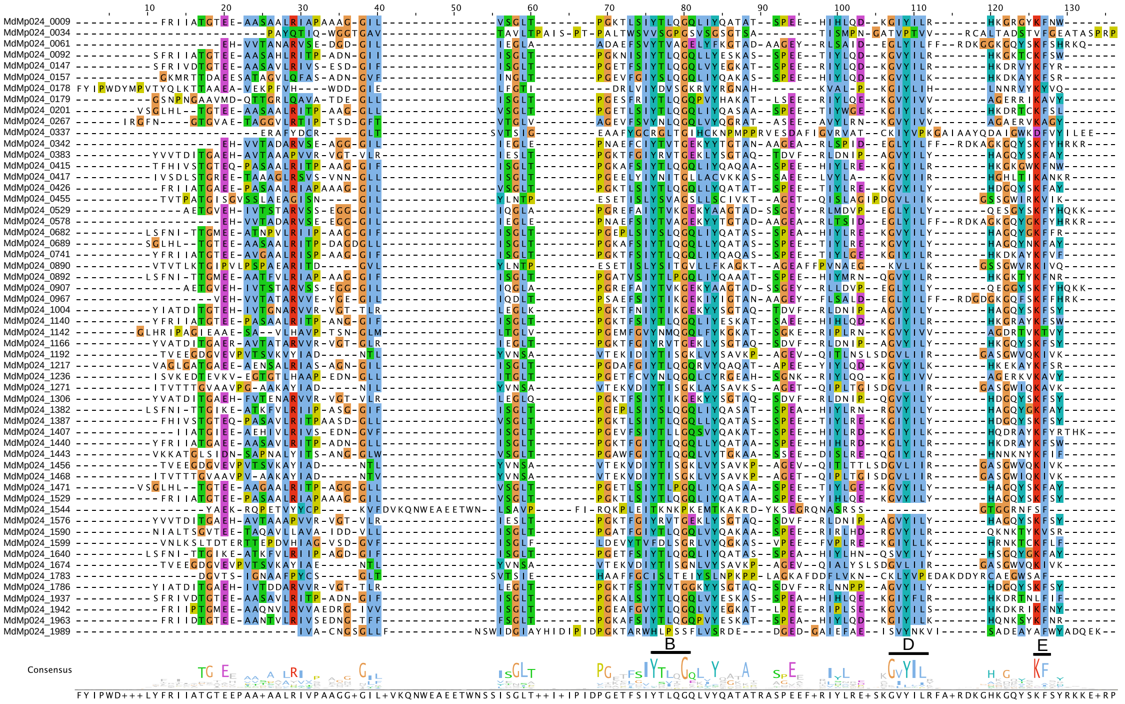

Figure S14. Alignment of the C-terminal 80 amino acid residues of immunoglobulin-like domain proteins in *Synergitannerella mixotrichae*. Three motifs (YxxxG, GxYIx, KF) were detected, which are similar to the motifs B (YDMNG), D (GxYxx), and E (KxxVx), respectively, previously reported in *Porphyromonas gingivalis* [51].

	388			391													404
**Hexose-6-phosphate:phosphate antiporter UhpT (*Escherichia coli*)**	D	G	I	K	G	T	F	A	Y	L	I	G	D	S	F	A	K
**Hexose-6-phosphate:phosphate antiporter UhpT (*Salmonella typhimurium*)**	D	G	I	K	G	T	F	A	Y	L	I	G	D	S	F	A	K
**Hexose-6-phosphate:phosphate antiporter UhpT (*Shigella flexneri*)**	D	G	I	K	G	T	F	A	Y	L	I	G	D	S	F	A	K
**Glycerol-3-phosphate transporter GlpT (*Escherichia coli*)**	A	G	F	T	G	L	F	G	Y	L	G	G	S	V	A	A	S
**Glycerol-3-phosphate transporter GlpT (*Bacillus subtilis*)**	A	G	L	T	G	F	F	G	Y	I	G	G	S	A	F	A	N
**Glycerol-3-phosphate transporter GlpT (*Haemophilus influenzae*)**	A	G	F	T	G	L	F	G	Y	L	G	G	T	V	S	A	S
**Membrane sensor protein UhpC (*Escherichia coli*)**	T	G	F	V	G	L	F	A	Y	L	G	A	S	L	A	G	W
**Membrane sensor protein UhpC (*Salmonella typhimurium*)**	T	G	F	V	G	L	F	A	Y	L	G	A	S	L	S	G	W
**TGRD_258 glucose 6-phosphate permease (*Ca.* Endomicrobiellum trichonymphae Rs-D17)**	C	G	F	T	G	M	F	G	Y	A	G	A	A	L	S	G	F
**Probable hexose phosphate transport protein (*Chlamydia pneumoniae*)**	S	G	F	T	G	W	F	A	Y	F	G	A	T	F	A	G	Y
**Phosphoglycerate transporter protein PgtP (*Salmonella typhimurium*)**	V	G	L	R	G	F	M	S	Y	I	F	G	A	S	L	G	T
**MdMp027_0318**	N	G	L	V	G	I	W	G	Y	A	S	V	I	V	T	G	W
**MdMp027_0408**	C	G	F	T	G	M	F	G	Y	L	G	A	F	L	S	G	V
**MdMp027_0733**	V	G	F	H	G	F	W	G	Y	L	S	V	I	A	T	G	W

Acidic AA: Asp(D), Glu(E)

Meduim hydrophilic AA: Cys(C), Met(M)

Small AA: Gly(G)

Basic AA: Arg(R), Lys(K), His(H)

Hydrophobic AA: Ala(A), Val(V), Ile(I), Leu(L), Phe(F), Pro(P), Trp(W), Tyr(Y)

Hydrophilic AA: Asn(N), Gln(Q), Ser(S), Thr(T)

Figure S15. Alignment of the 11th transmembrane site of UhpC/T homologs of *Endomicrobiellum mixotrichae* and other bacteria. MdMp027_0318, MdMp027_0408, and MdMp027_0733 are encoded by *E. mixotrichae.*

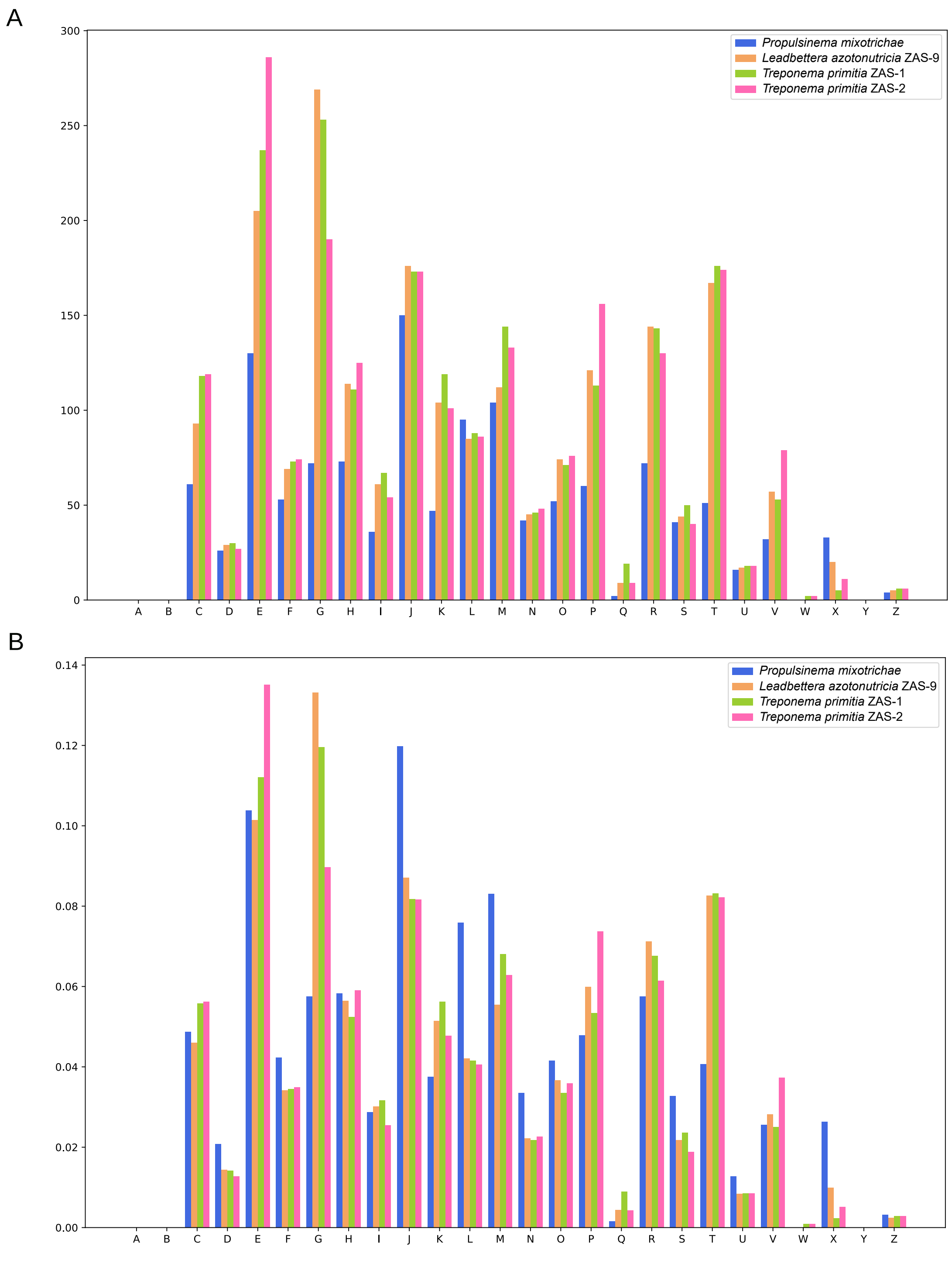

Figure S16. Comparison of number (A) and ratio (B) of genes classified into clusters of orthologous groups (COGs) between *Propulsinema mixotrichae* and its relatives. The categories denote the following functions: A) RNA processing and modification; B) chromatin structure and dynamics; C) energy production and conversion; D) cell cycle control, cell division, chromosome partitioning; E) amino acid transport and metabolism;

F) nucleotide transport and metabolism; G) carbohydrate transport and metabolism; H) coenzyme transport and metabolism; I) lipid transport and metabolism; J) translation, ribosomal structure and biogenesis; K) transcription; L) replication, recombination and repair; M) cell wall/membrane/envelope biogenesis; N) cell motility; O) posttranslational modification, protein turnover, chaperones; P) inorganic ion transport and metabolism; Q) secondary metabolites biosynthesis, transport and catabolism; R) general function prediction only; S) function unknown; T) signal transduction mechanisms; U) intracellular trafficking, secretion, and vesicular transport; V) defense mechanisms; W) extracellular structures; X) mobilome: prophages, transposons; Y) nuclear structure; Z) cytoskeleton.

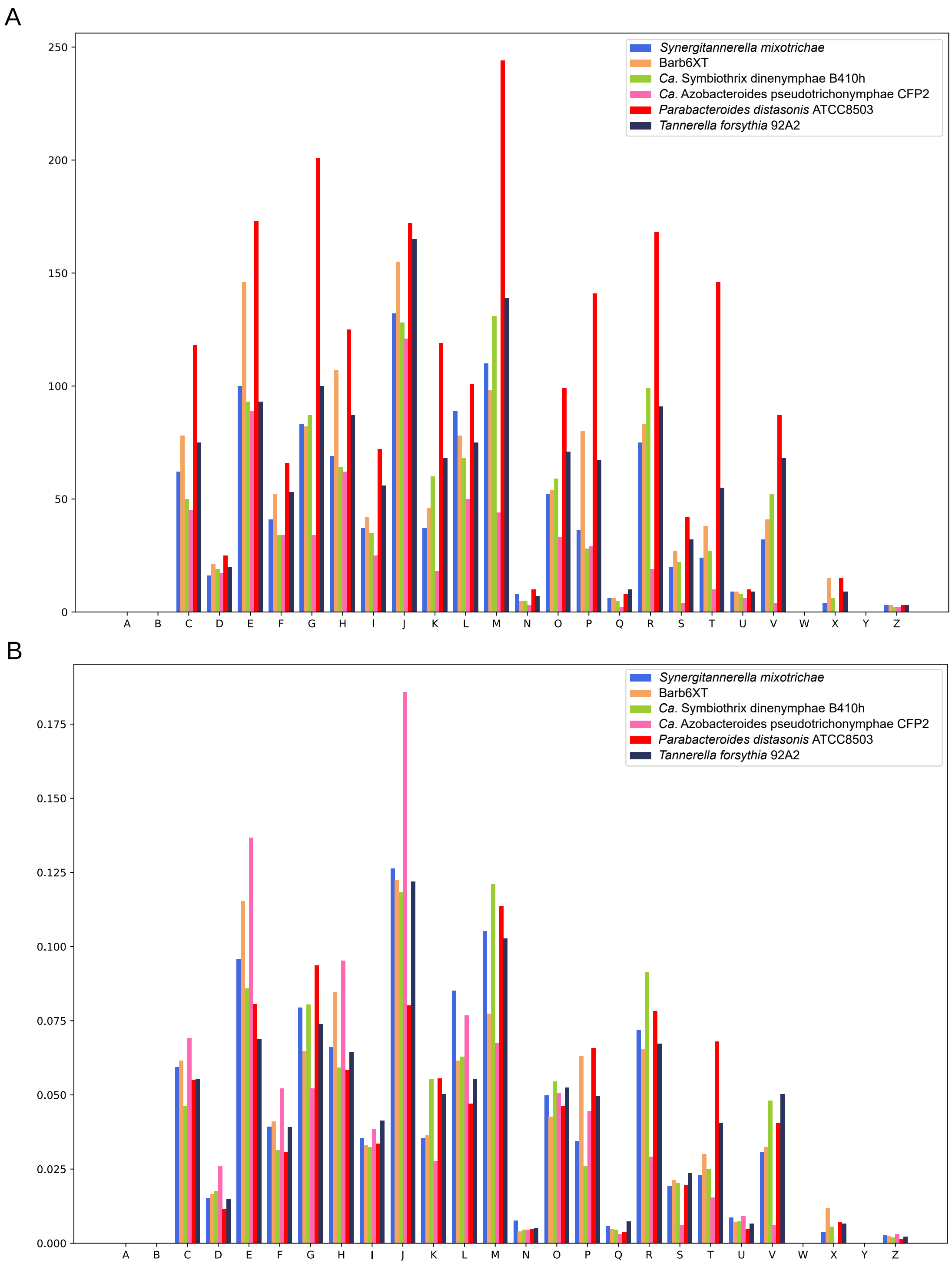

Figure S17. Comparison of number (A) and ratio (B) of genes classified into clusters of orthologous groups (COGs) between *Synergitannerella mixotrichae* and its relatives. See also the legend to Fig. S16.

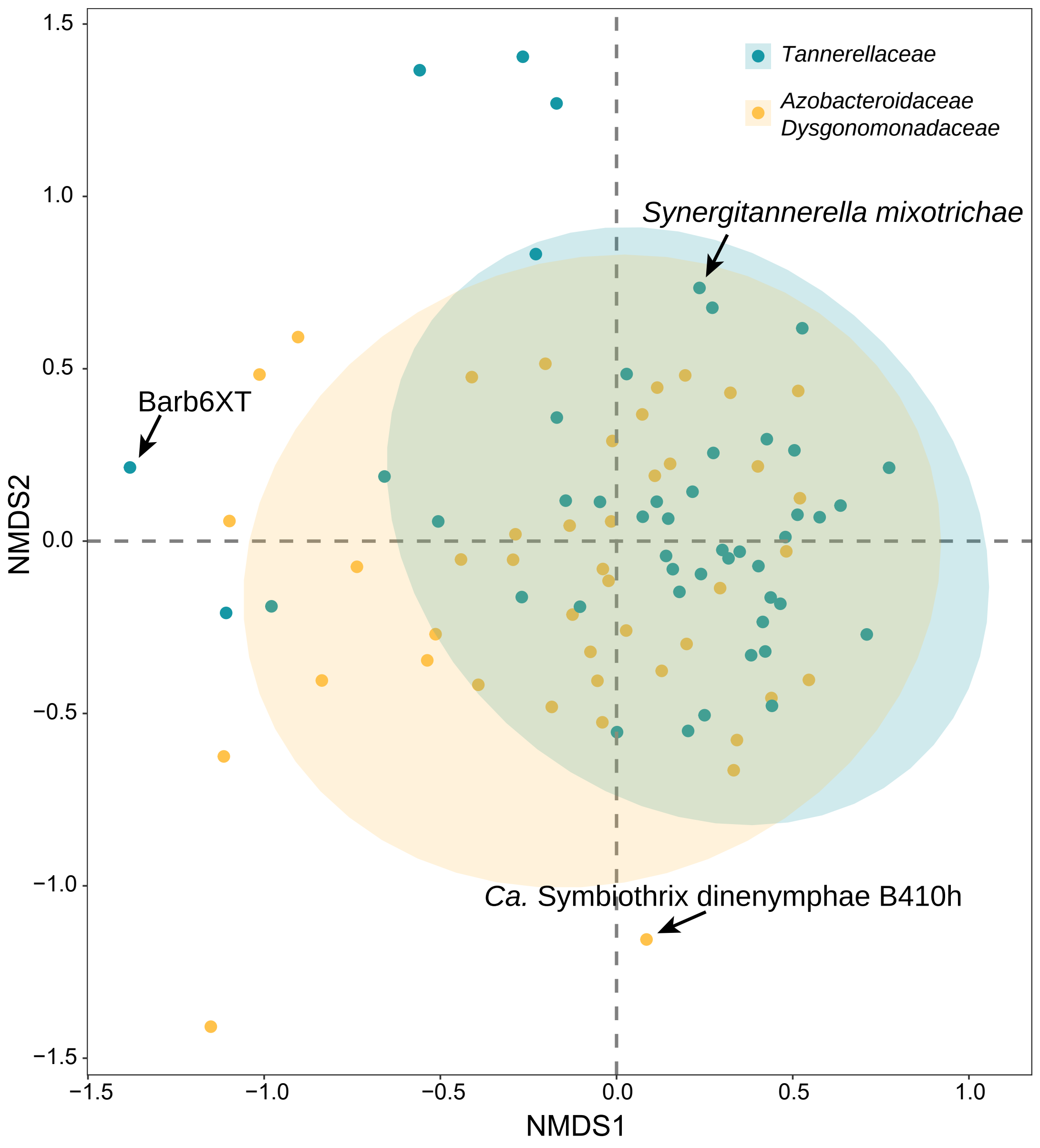

Figure S18. Non-metric multidimensional scaling analysis of wood decomposition-related glycoside hydrolase families in *Synergitannerella mixotrichae* and its relatives.

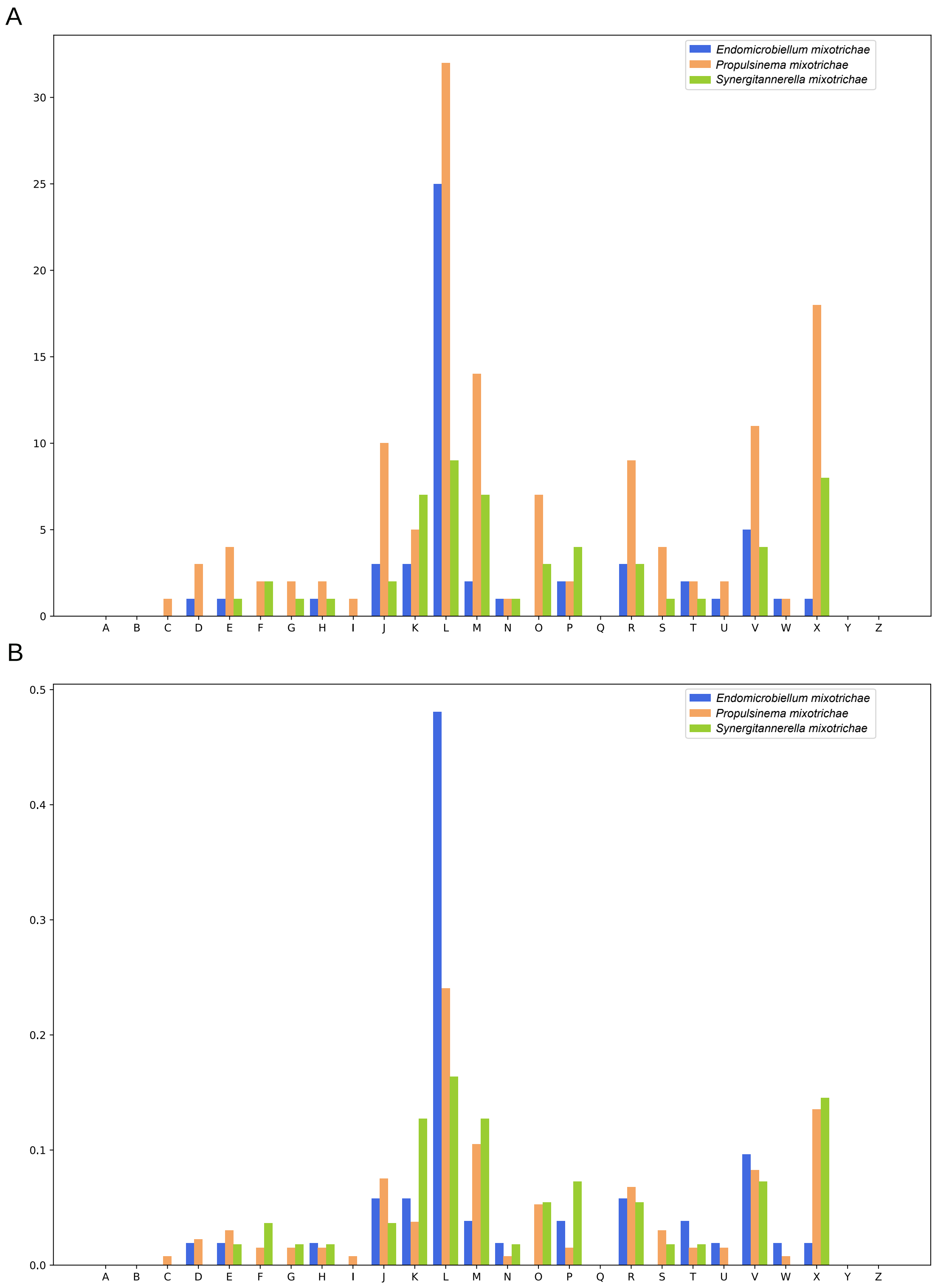

Figure S19. Comparison of number (A) and ratio (B) of pseudogenes classified into clusters of orthologous groups (COGs) among the bacterial symbionts of *Mixotricha paradoxa*. See also the legend to Fig. S16.
