# Supplementary material for: Functional division of labor in motility, lignocellulose digestion, and nitrogen metabolism revealed for the Mixotricha paradoxa holobiont
Source: ISME J. 2025 Aug 20;19(1):wraf178. doi: 10.1093/ismejo/wraf178 (PMC12483993; doi:10.1093/ismejo/wraf178)
Supplement: Supplementary_Text_wraf178(1) [file supplementary_text_wraf178(1).docx]

**Supplementary Text**

This Supplementary Text consists of Supplementary Methods, Supplementary Results, Legends to Supplementary Videos and Figures, and References to Supplementary Materials.

**Supplementary Methods**

**Phylogenetic analysis based on 16S rRNA genes**

The 16S rRNA genes of the genomes of *Propulsinema mixotrichae* and *Synergitannerella mixotrichae* were aligned with their respective reference sequences retrieved from the SILVA database r138.2 [1], the All-Species Living Tree Project LTP_10_2024 [2], and the NCBI non-redundant (nr) nucleotide database. The alignment was performed using MAFFT v7.490 [3] with default settings and trimmed using trimAL v1.5.rev0 [4] with the “-automated1” option.

**Whole genome amplification (WGA)**

*Mixotricha paradoxa* single-cell samples were transferred to a 0.2-mL PCR tube with 1 μL of sterilized double-distilled water (DDW). The samples were lysed with lysis buffer containing 0.4 M KOH, 100 mM dithiothreitol (DTT), and 10 mM ethylenediaminetetraacetic acid (Table S2), followed by brief centrifugation and incubation on ice for 10 min. After neutralization buffer (0.4 M HCl, 0.6 M Tris-HCl, pH 7.5) was added, the samples were mixed with reaction mixture, containing Reaction Buffer in the kit, 0.1 M DTT, DDW, 25 mM dNTP, 500 μM exonuclease-resistant random hexamer (New England Biolabs), and EquiPhi29 DNA polymerase (ThermoFisher Scientific) in the volumes shown in Table S2 for each sample. The reaction mixture was then incubated at 42°C for 3 h in a PCR thermal cycler. Finally, the mixture was incubated at 65°C for 10 min to inactivate the enzyme reaction. All regents and plasticware were UV-treated for decontamination.

**Second-round WGA, debranching, and DNA purification**

The second-round WGA for the best samples was performed using EquiPhi29 DNA polymerase in 50 or 100 µL reaction volume (Table S5). The reaction mixture containing Reaction Buffer, DDW, exonuclease-resistant random hexamer, and the first-round WGA products in the volumes shown in Table S5, was incubated in a PCR thermal cycler at 95°C for 3 min and then on ice for 3 to 5 min to stop the reaction. Subsequently, 100 mM DTT, 25 mM dNTP, and EquiPhi29 DNA polymerase were added (Table S5), and the reaction mixture was incubated at 45°C for 2 h and then inactivated at 65°C for 10 min.

These second-round WGA products were purified by ethanol precipitation and re-dissolved in 30 μL of TE buffer. Debranching and single-strand DNA digestion were performed using EquiPhi29 DNA polymerase and S1 nuclease (Takara Bio), respectively [5]. After processing, DNA was purified by ethanol precipitation.

**Sequencing on the MiSeq System and quality trimming**

For the first-round WGA samples, paired-end sequencing libraries were prepared using the Nextera XT DNA Library Preparation Kit (Illumina) for the cell membrane samples without DNase I treatment. The QIAseq FX DNA Library Kit (Qiagen) was used for the cell membrane samples with DNase I treatment and the cytoplasmic samples. For the second-round WGA samples, paired-end libraries were prepared using the TruSeq DNA PCR-Free High Throughput Library Prep Kit (Illumina). Sequencing was performed on a MiSeq System using the MiSeq Reagent Kit V3 (600 cycles). Adapter removal and quality trimming (Q < 30) of the MiSeq reads were conducted with cutadapt v2.10 [6] and prinseq v0.20 [7].

**Sequencing on the MinION platform and quality trimming**

The purified WGA products were fragmented using Covaris g-TUBE, followed by separation of DNA fragments by agarose gel electrophoresis. Fragments of 3–10 kbp (for cell membrane samples) and 2–10 kbp (for a cytoplasm sample) were excised and purified, using the Zymoclean Large Fragment DNA Recovery Kit (Zymo Research). Sequencing libraries were prepared using the SQK-LSK109 kit (Oxford Nanopore Technologies). The libraries were sequenced on a MinION platform (Oxford Nanopore Technologies) using the FLO-MIN111 flow cells (R10.3) for the cell membrane samples and the FLO-MIN106 flow cell (R9.4.1) for the cytoplasm sample, respectively. Basecalling for the MinION reads was performed using Guppy v1.1 alpha (https://community.nanoporetech.com), and adapters were removed using Porechop v0.2.4 (https://github.com/rrwick/Porechop). For the cell membrane samples, the MinION reads were quality trimmed using Fastp v0.21.0 [8] and FMLRC2 v0.1.2 [9]. For the cytoplasm sample, the MinION reads were quality trimmed using NanoFilt [10], and only reads of 3–10 kb were retained.

**Genome assembly and binning**

Sample selection

The quality-trimmed MiSeq reads were assembled using SPAdes v3.15.0 with the default --sc mode [11]. Genes coding for 16S rRNA were identified using RNAmmer v.1.2 [12]. Contigs were binned based on tetranucleotide frequency using the MyCC_2017 tool [13]. Bins containing 16S rRNA genes almost identical to mpsp15 (X89051) or B6 (AJ488195) were identified by BLASTn searches of the NCBI nr nucleotide database. The completeness and contamination rate of bins were evaluated using CheckM v1.1.3 [14] to select the best samples (Table S3).

Assembling genomes of *P. mixotrichae* and *S. mixotrichae*

The second-round WGA products of the selected samples were sequenced deeper on the MiSeq System using the MiSeq Reagent Kit V3 (600 cycle). The reads were quality trimmed, assembled, and binned as above. Contaminating contigs in the bin of *P. mixotrichae* or *S. mixotrichae* were identified using Contigs Annotation Tools [15] and were manually removed. Contigs in the decontaminated bins were scaffolded using SLR [16] with the quality-trimmed MinION reads. Gaps in scaffolds were closed using TGS-GapCloser [17], where possible. The quality-trimmed MiSeq reads and MinION reads were mapped onto the scaffolds using BBMap v38.96 [18] and minimap2 [19], respectively. The mapped MiSeq and MinION reads were collected and hybrid-assembled [20] together using SPAdes v.3.15.0 with default settings. Finally, contaminating contigs were removed again as above.

Assembling genome of *Endomicrobiellum mixotrichae*

Of the quality trimmed MinION reads, 50% were randomly extracted using SeqKit [21] and assembled with Flye v2.8 [22], resulting in a circular contig. The quality trimmed MiSeq reads were then mapped onto the circular contig using Bowtie2 [23] with the “very sensitive” mode, and the contig was polished one time with Pilon [24] with the “SNP/indel only” mode. Both MiSeq and MinION reads were re-mapped to the polished circular contig using Bowtie2 and Minimap2, respectively, to verify the accuracy of assembly. Visual inspection of the mapping results identified two regions with no MiSeq read coverage, which were examined through PCR and Sanger sequencing. The respective ambiguous regions were then replaced with the sequences obtained using the Sanger method.

**Phylogenomic analyses**

We collected genome sequences assigned to *Breznakiellaceae* or *Tannerellaceae/Porphyromonadaceae/Azobacteroidaceae/Dysgonomonadaceae* from GTDB r220 [25] and recent large-scale analyses of termite-gut microbiomes [26–28], using GTDB-Tk v2.1.0 [29]. Conserved single-copy marker genes for the phylogenomic analysis were extracted using CheckM for *P. mixotrichae* and GToTree v1.8.2 [30] for *S. mixotrichae*. Sequences were concatenated and aligned using MAFFT v7.471 and trimmed with TrimAL v1.4rev22. Maximum-likelihood trees were constructed using IQ-TREE v1.6.12.

**Identification and phylogenetic analysis of *nifHDK***

Genes for NifHDK were identified using hmmscan v2.3.2 against profiles PF00142.22, TIGR01282.1, and TIGR01286.1 [31]. The dataset for phylogenetic analysis of NifH homologs was collected following a previous study [32] (Table S13). For detailed phylogenetic analyses of NifH1 and NifH2 of *P. mixotrichae*, the top 100 sequences showing the highest homology to each sequence in BLASTp searches of the NCBI nr protein database were collected (Table S14). The alignment was performed using MAFFT v7.490 with default settings and trimmed using trimAL v1.5.rev0 with the “-automated1” option. Maximum likelihood trees were constructed using IQ-TREE v1.6.12 and 1,000 ultrafast bootstrap and SH-aLRT test replicates [33]. Sequence alignments were visualized using Jalview [34]. Phylogenetic trees were visualized using iTOL [35].

**Library preparation for RNA-seq**

The size distribution of cDNA sequence from the single and three-cell *M. paradoxa* samples was checked using an Agilent 2100 Bioanalyzer, and the concentration was quantified using a Qubit Fluorometer (ThermoFisher Scientific). Sequence libraries were prepared using the Nextera XT DNA Library Preparation Kit and then subjected to agarose gel electrophoresis for size selection. Fragments of 550–700 bp were extracted using the MinElute Gel Extraction Kit (Qiagen) and quantified using the Qubit Fluorometer with the KAPA Library Quantification Kit (Roche Diagnostic).

**Quality trimming of reads, filtering of contigs, and functional annotation in transcriptomics of *M. paradoxa***

Adapters were trimmed using cutadapt v3.7, and low-quality bases and poly-A/T tails were removed with prinseq v0.20.4. The quality-trimmed reads were assembled using Trinity v2.6.6 with default settings (https://github.com/trinityrnaseq/). Open reading frames (ORFs) were predicted with TransDecoder v5.7.0 (https://github.com/TransDecoder/TransDecoder) using BlastP searches of the uniprot database (uniprot_sprot, v2022-04) and hmmsearch of Pfam database (Pfam-A, v2021-03-19).

We filtered contigs to exclude those identified as 1) noncoding RNAs, 2) potential bacterial sequences, and 3) potential mis-assemblies. Noncoding RNAs were annotated using structRNAfinder [36] and were excluded. To identify bacterial sequences, we mapped all MiSeq and MinION reads generated from the WGA products of the bacterial communities associated with *M. paradoxa* (Table S4), to the contigs using BBMap v38.96, and those aligned with the bacterial reads were eliminated. A subset of the contigs (7.2% and 5.5% of total contigs in the single and three-cell *M. paradoxa* assemblies, respectively) contained multiple putative ORFs. We decided to eliminate these contigs, considering the possibility of mis-assemblies or contamination of bacterial transcripts

Functional annotation was performed using PANNZER2 [37]. Transcripts for CAZymes were identified using dbCAN v4.0.0. ORFs were classified into KEGG Orthology (KO) categories using eggNOG-mapper v2 [38] and the KEGG automatic annotation server (KAAS) [39]. Statistical analysis and visualization were performed using TBtools-II [40]. Metabolic pathways were reconstructed using the KAAS and KEGG Mapper. Redundant glycoside hydrolase (GHs) transcripts in the single and three-cell *M. paradoxa* samples were clustered using CD-HIT [41] with a 95% similarity threshold (Fig. 5B and Table S10).

**Phylogenetic analysis of tryptophan synthase TrpB**

To infer the phylogenetic positions of TrpB homologs detected in the *M. paradoxa* transcriptomes, TrpB sequences were collected from previously published transcriptome data generated from parabasalid flagellates. In addition, the top 50 sequences showing the highest homology to each TrpB sequence from the parabasalid flagellates were identified and retrieved by BLASTp searches of the NCBI nr protein database. Duplicated reference sequences were removed using SeqKit (Table S15). Alignment, trimming, and tree construction followed the same methods for the phylogenetic analysis of NifH.

**Relative abundance of genome fragments of bacterial symbionts**

To calculate the relative abundance of genome fragments of *P. mixotrichae*, *S. mixotrichae*, and *E. mixotrichae* in each sequence dataset generated from the first-round WGA samples, the quality-trimmed MiSeq reads were mapped onto those bacterial genome assemblies using BBMap v38.96 (Table S4). The source of unmapped reads was predicted using MMseqs2 v13-45111 [42] (Table S4).

**Supplementary Results**

**Identification of two nitrogen reductase gene sets**

In the *P. mixotrichae* genome, two nitrogenase reductase gene homologs (*nifH1* and *nifH2*) assigned to the NifH group II were identified (Figs. S10 and S11). The *nifH1* gene formed a typical FeMo-dependent nitrogenase operon (Fig. S12A), whereas *nifH2* is located distantly from its counterparts, *nifD2 and nifK2* (Fig. S12B). NifH2 may have a different function other than nitrogen fixation [43]. The presence of both *nifH1* and *nifH2* was confirmed in the *P. mixotrichae* genomes from other six samples (Tables S3 and S9).

**COG functional categories with reduced gene number in *P. mixotrichae***

In the *P. mixotrichae* genome, several COG functional categories exhibited prominent reduction in the number of genes in comparison to its free-swimming relatives (Fig. S16A). The reduced categories include (C) “energy production and conversion”, (E) “amino acid transport and metabolism”, (G) “carbohydrate transport and metabolism”, (P) “inorganic ion transport and metabolism”, and (T) “signal transduction mechanisms” (Fig. S16A).

**Identification of type IV secretion system in *P. mixotrichae***

In the *P. mixotrichae* genome, genes for two ATPase subunits (VirB4 and VirB11) of the type IV secretion system (T4SS) were not found, and the last ATPase gene *virD4* was found to be corrupted. However, intact genes coding for these three ATPases were detected in the genomes of *P. mixotrichae* from other first-round WGA samples; thus, the absence and corruption of these genes are possibly attributable to the genome incompleteness. We therefore consider that the T4SS is functional in *P. mixotrichae.*

**Classification of pseudogenes based on clusters of orthologous genes (COG) in the bacterial symbionts of *M. paradoxa***

The pseudogenes of the three symbiotic bacteria were classified into COG functional categories (Fig. S19). Pseudogenes were prominently accumulated in category (L) “replication, recombination and repair” especially in the genomes of *P. mixotrichae* and *E. mixotrichae*. The proportion of pseudogenes in category (L) was particularly high in *E. mixotrichae*, and, in contrast, that in category (X) “mobilome: prophages, transposons” was very low. This trait in *E. mixotrichae* is commonly observed among obligate intracellular bacterial symbionts with a reduced genome size [44].

**Description of *Propulsinema* gen. nov**

*Propulsinema* [Pro.pul.si.ne’ma. L. neut. n. *propulsare*, to propel, push forward; Gr. neut. n. *nema*, a thread; N.L. neut. n. *Propulsinema*, a thread-like bacterium associated with propulsion or motility]. This genus includes a motile, thread-like bacterium that colonizes the cell surface of a flagellated protist in the gut of a termite species and provides the locomotive force to propel the host cell. The type species is *Propulsinema mixotrichae*, which remains uncultured. The average nucleotide identities (ANIs) between *P. mixotrichae* and the type species of other genera in *Breznakiellaceae* (i.e., *Gracilinema caldarium*, *Breznakiella homolactica*, and *Leadbettera azotonutricia*) are below 70%.

**Description of *Synergitannerella* gen. nov**

*Synergitannerella* [Sy.ner.gi.tan.ne.rel’la. Gr. pref. *synergi-*, cooperation, synergy; N.L. fem. n. *Tannerella*, taxonomic name of a bacterial genus; N.L. fem. n. *Synergitannerella*, a bacterium associated with cooperative interactions and phylogenetically related to *Tannerella*]. This genus includes a bacterium that colonizes the cell surface of a flagellated protist in the gut of a termite species. The type species is *Synergitannerella mixotrichae*, which remains uncultured. The ANIs between *S. mixotrichae* and the type species of other genera in *Tannerellaceae* (i.e., *Tannerella forsythia* and *Parabacteroides distasonis*) are below 70%.

**Description of *Propulsinema mixotrichae* sp. nov**

*Propulsinema mixotrichae* (mix.o.tri’chae. N.L. fem. n. *mixotrichae*, of *Mixotricha*, a genus of flagellated protists, referring to the host). The spiral-shaped bacteria measure 0.5–7.0 μm in length and 0.1–0.3 μm in diameter. They are motile and obligately colonize almost the entire cell surface, except for the posterior region, of *Mixotricha paradoxa* in the gut of *Mastotermes darwiniensis*. The assignment is based on the 16S rRNA gene (LC852377), the draft genome (SAMD00846338), and hybridization with the 16S rRNA-targeted probe MdMp-014-133 (5′-TCCCCATCCTCTAGGGCA-3′). The species comprises all bacteria with >95% ANIs to the type genome.

**Description of *Synergitannerella mixotrichae* sp. nov**

*Synergitannerella mixotrichae* (mix.o.tri’chae. N.L. fem. n. *mixotrichae*, of *Mixotricha*, a genus of flagellated protist, referring to the host). The bacteria are rod shaped with dimensions of 0.5–2.0 μm by 0.3–1.0 μm. They are non-motile and obligately colonize the cell surface of *Mixotricha paradoxa* in the gut of *Mastotermes darwiniensis*. The assignment is based on the 16S rRNA gene (LC852376), the draft genome (SAMD00846339), and hybridization with the 16S rRNA-targeted probe MdMp-bact197 (5′-TCCTCCGGCAATTCCTCTT-3′). The species comprises all bacteria with >95% ANIs to the type genome.

**Description of *Endomicrobiellum mixotrichae* sp. nov**

*Endomicrobiellum mixotrichae* (mix.o.tri’chae. N.L. fem. n. *mixotrichae*, of *Mixotricha*, a genus of flagellated protist, referring to the host). The bacteria are rod shaped with dimensions of 1.0–2.0 μm by 0.4–1.0 μm. The bacteria are non-motile and obligately colonize the cytoplasm of *Mixotricha paradoxa* in the gut of *Mastotermes darwiniensis*. The assignment is based on the 16S rRNA gene (LC852374), the complete genome (SAMD00846340), and hybridization with the 16S rRNA-targeted probe MdMp-027-644 (5′-CCCAAACTCAAGCTGAAC-3′). The species comprises all bacteria, including the MAG Md513_bin60 (99.4% ANI), with >95% ANIs to the type genome.

**Legends to Supplementary Videos and Figures**

**Video S1. Swimming styles of *Mixotricha paradoxa* (top) and *Deltotrichonympha operculata* (bottom).** Video was captured under a differential interference contrast microscope (Leica AM6000). The gut content of a worker of *Mastotermes darwiniensis* was suspended in solution U. *Mixotricha paradoxa* swims relatively slowly using its ectosymbiotic spirochetes, compared to the parabasalid *D. operculata*, which swims faster using its own numerous flagella.

**Video S2. Movement of ectosymbiotic spirochetes covering the surface of *Mixotricha paradoxa*.** Video was captured under a differential interference contrast microscope (Leica AM6000).

**Figure S1. Detection of *Propulsinema mixotrichae* and *Synergitannerella mixotrichae* on the cell surface of *Mixotricha paradoxa* by fluorescence in situ hybridization (FISH).** (A) Phase-contrast image of *M. paradoxa*. (B) DAPI stain (blue). White arrows indicate the boundary of area densely covered by ectosymbionts. The posterior area without ectosymbionts is likely used for phagocytosis of wood particles. (C) Magnified phase-contrast image showing short spirochetes covering the host cell surface and a long spirochete indicated by black arrow. (D–F) Epifluorescence images. (D) Detection of *S. mixotrichae* using probe MdMp-bact197 (6FAM-labeled, green) (Table S1). White arrows indicate the boundary of area densely covered by *S. mixotrichae*. Yellow fragments are wood particles phagocytosed by *M. paradoxa*. (E) Detection of *P. mixotrichae* using probe MdMp-014-133 (Texas red-labeled, red) (Table S1). White arrows indicate the boundary of area densely covered by *P. mixotrichae*. (F) Magnified view of panel E, corresponding to the region of panel C. Only short spirochetes were detected by the FISH analysis. Bars: (A, B, D, E) 50 μm; (C, F) 10 μm.

**Figure S2. Transmission electron micrographs of *Mixotricha paradoxa* and its symbiotic bacteria.** (A) Insertion of the anterior end of *Propulsinema mixotrichae* into the invagination of the cell surface of *M. paradoxa*. (B) Food vacuole of *M. paradoxa* containing several bacterial cells. (C) Food vacuole-like structure containing several bacterial cells (black arrow). White arrow indicates a food vacuole filled with a wood fragment. (D) Bacteria resembling *Endomicrobiellum mixotrichae* (black arrow, Fig. 1H) are observed within a food vacuole-like structure, alongside wood fragments (gray arrow) and remnants of digested bacterial cells (white arrow). (E) Magnified image of the area indicated in panel C. Endoplasmic flagellum (black arrow), characteristic to spirochetes, is visible in several bacterial cells [45]. Bars: (A, E) 100 nm; (B, C, D) 500 nm.

**Figure S3. Phylogenetic position of *Propulsinema mixotrichae* based on 16S rRNA gene.** Maximum-likelihood tree was constructed using the TIM3+F+R4 model based on 1,462 aligned positions, with *Spirochaeta isovalerica* (FR749931) and *Spirochaeta cellobiosiphila* (EU448140) used as outgroups. Host termite or cockroach species are shown in parentheses. Highly supported nodes (ultrafast bootstrap support ≥ 95%, SH-aLRT ≥ 80%, 1,000 replicates) are indicated with a closed circle. Taxonomy was based on GTDB.

**Figure S4. Phylogenetic position of *Synergitannerella mixotrichae* based on 16S rRNA gene.** Maximum-likelihood tree was constructed using the GTR+F+R4 model based on 1,352 aligned positions, with *Capnocytophaga granulosa* (MW555275) and *Flavobacterium arcticum* (KU529277) used as outgroups. Host termite or cockroach species are shown in parentheses. Highly supported nodes (ultrafast bootstrap support ≥ 95%, SH-aLRT ≥ 80%, 1,000 replicates) are indicated with a closed circle. Taxonomy was based on GTDB.

**Figure S5. Comparison of transcriptome or genome completeness between *Mixotricha paradoxa* and other parabasalid flagellates.** Transcriptomes of four parabasalid flagellates (i.e., *Cononympha leidyi*, *Holomastigotoides hartmanni*, *Holomastigotoides minor*, *Pseudotrichonympha grassii*) in the gut of the termite *Coptotermes formosanus* were obtained from a previous study [46]. The genomes of *Tritrichomonas foetus* and *Trichomonas vaginalis* were retrieved from TrichDB release 57 (https://trichdb.org/trichdb/app). The completeness was estimated using BUSCO v4.0.6 with the dataset “eukaryota_odb10.2019-11-2”.

**Figure S6. Phylogenetic positions of TrpB expressed by parabasalid flagellates.** A maximum-likelihood tree was constructed using the LG+I+G4 model based on 454 aligned positions. Transcripts of *trpB* were detected both in the single-cell and three-cell *M. paradoxa* samples and were phylogenetically closest to TrpB expressed by *Pseudotrichonympha grassii* in the gut of *Coptotermes formosanus* [46]. Highly supported nodes (ultrafast bootstrap support ≥ 95%, SH-aLRT ≥ 80%, 1,000 replicates) are indicated with a closed circle.

**Figure S7. Transcriptome profile of *Mixotricha paradoxa* and other parabasalid flagellates.** The functional categories were assigned based on KEGG orthology. Abundance was calculated as transcripts per million and subjected to row normalization. The data for *M. paradoxa* (Mp) are mean values of the single and three-cell samples. The data for other flagellate species are mean values of three samples reported previously. Cl: *Cononympha leidyi*; Hh: *Holomastigotoides hartmanni*; Hm: *Holomastigotoides minor*; Pg: *Pseudotrichonympha grassii* (DRR1852229 to DRR185224) [46]; Tfo: *Tritrichomonas foetus* (ERR4352424, ERR4398931, and ERR4398932); Tva: *Trichomonas vaginalis* (SRR2132589 to SRR2132591).

**Figure S8. Glycoside hydrolase (GH) genes expressed by *Mixotricha paradoxa* and other parabasalid flagellates.** Abundance was calculated as transcripts per million (TPM) and subjected to log_10_(x+1) transformation for display in the heatmap. The vertical axis includes all GH families expressed by *M. paradoxa* as well as the top 10 GH families expressed by the other parabasalid species. The order of GH families is based on TPM in *M. paradoxa* (Mp). The data for *M. paradoxa* are mean values of the single and three-cell samples. The data for other flagellate species are mean values of three samples reported previously. Cl: *Cononympha leidyi*; Hh: *Holomastigotoides hartmanni*; Hm: *Holomastigotoides minor*; Pg: *Pseudotrichonympha grassii* (DRR1852229 to DRR185224) [46]; Tfo: *Tritrichomonas foetus* (ERR4352424, ERR4398931, and ERR4398932); Tva: *Trichomonas vaginalis* (SRR2132589 to SRR2132591).

**Figure S9. GC content and genome size of *Propulsinema mixotrichae* (A) and *Synergitannerella mixotrichae* (B) in comparison with their respective relatives.** The metagenome-assembled genome of “*Candidatus* Ordinivivax streblomastigis”, which is an ectosymbiont of the oxymonad flagellate *Streblomastix strix* [47] was not included in panel B due to its low genome completeness (< 80%).

**Figure S10. Phylogenetic assignment of two NifH homologs of *Propulsinema mixotrichae* into NifH Groups I–V.** A maximum-likelihood tree was constructed using the LG+G4 model based on 221 aligned amino acid positions. Highly supported nodes (ultrafast bootstrap support ≥ 95%, SH-aLRT ≥ 80%, 1,000 replicates) are indicated with a closed circle. The NifH groups have been proposed by a previous study [48].

**Figure S11. Phylogenetic positions of two NifH homologs of *Propulsinema mixotrichae* within NifH Group II.** (A) Phylogenetic positions of NifH1 and NifH2 of *P. mixotrichae* among the whole Group II sequences. (B) Detailed position of NifH1 of *P. mixotrichae*. (C) Detailed position of NifH2 of *P. mixotrichae*. A maximum-likelihood tree was constructed using the LG+R5 model based on 272 aligned amino acid positions. Highly supported nodes (ultrafast bootstrap support ≥ 95%, SH-aLRT ≥ 80%, 1,000 replicates) are indicated with a closed circle.

**Figure S12. Structures of *nif* gene clusters.** (A) Gene cluster containing *nifH1* of *Propulsinema mixotrichae*. It forms a typical FeMo-dependent nitrogenase operon with *nifHDKENB* with nitrogen regulatory protein PII genes, *nifI1* and *nifI2*. (B) Gene cluster containing *nifH2* of *P. mixotrichae* and clusters with its homologs in reference metagenome-assembled genomes from termite guts. The *nifH2* gene do not form an operon with *nifDK*, and its homologs also do not constitute a typical *nif*-operon for nitrogen fixation.

**Figure S13. Schematic representation of the type IX secretion system (T9SS) encoded by the genome of *Synergitannerella mixotrichae*.** A substrate of T9SS generally contains both a signal peptide (SP) and a conserved C-terminal domain (CTD). The SP enables recognition by the SEC system for transport to the periplasm, after which the CTD is recognized by the T9SS. The substrate is transferred to the PorV shuttle via the SprA translocon and then transported to the PorQUZ complex. Finally, the PorU sortase cleaves the CTD, releasing the protein into the medium or anchoring it to the cell surface. The schematic diagram is based on previous studies [49,50].

**Figure S14. Alignment of the C-terminal 80 amino acid residues of immunoglobulin-like domain proteins in *Synergitannerella mixotrichae*.** Three motifs (YxxxG, GxYIx, KF) were detected, which are similar to the motifs B (YDMNG), D (GxYxx), and E (KxxVx), respectively, previously reported in *Porphyromonas gingivalis* [51]*.*

**Figure S15. Alignment of the 11th transmembrane site of UhpC/T homologs of *Endomicrobiellum mixotrichae* and other bacteria.** MdMp027_0318, MdMp027_0408, and MdMp027_0733 are encoded by *E. mixotrichae.*

**Figure S16. Comparison of number (A) and ratio (B) of genes classified into clusters of orthologous groups (COGs) between *Propulsinema mixotrichae* and its relatives.** The categories denote the following functions: A) RNA processing and modification; B) chromatin structure and dynamics; C) energy production and conversion; D) cell cycle control, cell division, chromosome partitioning; E) amino acid transport and metabolism; F) nucleotide transport and metabolism; G) carbohydrate transport and metabolism; H) coenzyme transport and metabolism; I) lipid transport and metabolism; J) translation, ribosomal structure and biogenesis; K) transcription; L) replication, recombination and repair; M) cell wall/membrane/envelope biogenesis; N) cell motility; O) posttranslational modification, protein turnover, chaperones; P) inorganic ion transport and metabolism; Q) secondary metabolites biosynthesis, transport and catabolism; R) general function prediction only; S) function unknown; T) signal transduction mechanisms; U) intracellular trafficking, secretion, and vesicular transport; V) defense mechanisms; W) extracellular structures; X) mobilome: prophages, transposons; Y) nuclear structure; Z) cytoskeleton.

**Figure S17. Comparison of number (A) and ratio (B) of genes classified into clusters of orthologous groups (COGs) between *Synergitannerella mixotrichae* and its relatives.** See also the legend to Fig. S16.

**Figure S18. Non-metric multidimensional scaling analysis of wood decomposition-related glycoside hydrolase families in *Synergitannerella mixotrichae* and its relatives.**

**Figure S19. Comparison of number (A) and ratio (B) of pseudogenes classified into clusters of orthologous groups (COGs) among the bacterial symbionts of *Mixotricha paradoxa*.** See also the legend to Fig. S16.

**References to Supplementary Materials**

1. Pruesse E, Quast C, Knittel K *et al.* SILVA: a comprehensive online resource for quality checked and aligned ribosomal RNA sequence data compatible with ARB. *Nucleic Acids Res* 2007;**35**:7188–96.

2. Ludwig W, Viver T, Westram R *et al*. Release LTP_12_2020, featuring a new ARB alignment and improved 16S rRNA tree for prokaryotic type strains. *Syst Appl Microbiol* 2021;**44**:e126218.

3. Katoh K, Standley DM. MAFFT multiple sequence alignment software version 7: Improvements in performance and usability. *Mol Biol Evol* 2013;**30**:772–80.

4. Capella-Gutiérrez S, Silla-Martínez JM, Gabaldón T. trimAl: a tool for automated alignment trimming in large-scale phylogenetic analyses. *Bioinformatics* 2009;**25**:1972–3.

5. Zhang K, Martiny AC, Reppas NB *et al*. Sequencing genomes from single cells by polymerase cloning. *Nat Biotechnol* 2006;**24**:680–6.

6. Martin M. Cutadapt removes adapter sequences from high-throughput sequencing reads. *EMBnet J* 2011;**17**:e10.

7. Schmieder R, Edwards R. Quality control and preprocessing of metagenomic datasets. *Bioinformatics* 2011;**27**:863–4.

8. Chen S, Zhou Y, Chen Y *et al*. Fastp: an ultra-fast all-in-one FASTQ preprocessor. *Bioinformatics* 2018;**34**:i884–90.

9. Wang JR, Holt J, McMillan L *et al*. FMLRC: hybrid long read error correction using an FM-index. *BMC Bioinformatics* 2018;**19**:50.

10. De Coster W, D’Hert S, Schultz DT *et al*. NanoPack: visualizing and processing long-read sequencing data. *Bioinformatics* 2018;**34**:2666–9.

11. Bankevich A, Nurk S, Antipov D *et al*. SPAdes: a new genome assembly algorithm and its applications to single-cell sequencing. *J Comput Biol* 2012;**19**:455–77.

12. Lagesen K, Hallin P, Rødland EA *et al*. RNAmmer: consistent and rapid annotation of ribosomal RNA genes. *Nucleic Acids Res* 2007;**35**:3100–8.

13. Lin HH, Liao YC. Accurate binning of metagenomic contigs via automated clustering sequences using information of genomic signatures and marker genes. *Sci Rep* 2016;**6**:e24175.

14. Parks DH, Imelfort M, Skennerton CT *et al*. CheckM: Assessing the quality of microbial genomes recovered from isolates, single cells, and metagenomes. *Genome Res* 2015;**25**:1043–55.

15. von Meijenfeldt FAB, Arkhipova K, Cambuy DD *et al*. Robust taxonomic classification of uncharted microbial sequences and bins with CAT and BAT. *Genome Biol* 2019;**20**:e217.

16. Luo J, Lyu M, Chen R *et al*. SLR: a scaffolding algorithm based on long reads and contig classification. *BMC Bioinformatics* 2019;**20**:e539.

17. Xu M, Guo L, Gu S *et al*. TGS-GapCloser: a fast and accurate gap closer for large genomes with low coverage of error-prone long reads. *GigaScience* 2020;**9**:1–11.

18. Bushnell B. BBMap: a fast, accurate, splice-aware aligner. In: *the 9th Annual Genomics of Energy & Environment Meeting*, Walnut Creek, CA, 2014.

19. Li H. Minimap2: pairwise alignment for nucleotide sequences. *Bioinformatics* 2018;**34**:3094–100.

20. Antipov D, Korobeynikov A, McLean JS *et al*. HybridSPAdes: an algorithm for hybrid assembly of short and long reads. *Bioinformatics* 2016;**32**:1009–15.

21. Shen W, Le S, Li Y *et al*. SeqKit: a cross-platform and ultrafast toolkit for FASTA/Q file manipulation. *PLoS One* 2016;**11**:e0163962.

22. Kolmogorov M, Yuan J, Lin Y *et al*. Assembly of long, error-prone reads using repeat graphs. *Nat Biotechnol* 2019;**37**:540–6.

23. Langmead B, Salzberg SL. Fast gapped-read alignment with Bowtie 2. *Nat Methods* 2012;**9**:357–9.

24. Walker BJ, Abeel T, Shea T *et al*. Pilon: an integrated tool for comprehensive microbial variant detection and genome assembly improvement. *PLoS One* 2014;**9**:e112963.

25. Parks DH, Chuvochina M, Chaumeil PA *et al*. A complete domain-to-species taxonomy for Bacteria and Archaea. *Nat Biotechnol* 2020;**38**:1079–86.

26. Hervé V, Liu P, Dietrich C *et al*. Phylogenomic analysis of 589 metagenome-assembled genomes encompassing all major prokaryotic lineages from the gut of higher termites. *PeerJ* 2020;**8**:e8614.

27. Arora J, Kinjo Y, Šobotník J *et al*. The functional evolution of termite gut microbiota. *Microbiome* 2022;**10**:e78.

28. Salgado JFM, Hervé V, Vera MAG *et al*. Unveiling lignocellulolytic potential: a genomic exploration of bacterial lineages within the termite gut. *Microbiome* 2024;**12**:e201.

29. Chaumeil PA, Mussig AJ, Hugenholtz P *et al*. GTDB-Tk: a toolkit to classify genomes with the genome taxonomy database. *Bioinformatics* 2020;**36**:1925–7.

30. Lee MD. GToTree: a user-friendly workflow for phylogenomics. *Bioinformatics* 2019;**35**:4162–4.

31. Finn RD, Clements J, Eddy SR. HMMER web server: interactive sequence similarity searching. *Nucleic Acids Res* 2011;**39**:W29–37.

32. Morimoto Y, Uesaka K, Fujita Y, Yamamoto H. A nitrogenase-like enzyme is involved in the novel anaerobic assimilation pathway of a sulfonate, isethionate, in the photosynthetic bacterium *Rhodobacter capsulatus*. *mSphere* 2024;**9**:e00498-24.

33. Nguyen LT, Schmidt HA, von Haeseler A *et al*. IQ-TREE: a fast and effective stochastic algorithm for estimating maximum-likelihood phylogenies. *Mol Biol Evol* 2015;**32**:268–74.

34. Waterhouse AM, Procter JB, Martin DMA *et al*. Jalview version 2—a multiple sequence alignment editor and analysis workbench. *Bioinformatics* 2009;**25**:1189–91.

35. Letunic I, Bork P. Interactive tree of life (iTOL) v5: an online tool for phylogenetic tree display and annotation. *Nucleic Acids Res* 2021;**49**:W293–6.

36. Arias-Carrasco R, Vásquez-Morán Y, Nakaya HI *et al*. StructRNAfinder: an automated pipeline and web server for RNA families prediction. *BMC Bioinformatics* 2018;**19**:e55.

37. Törönen P, Holm L. PANNZER—a practical tool for protein function prediction. *Protein Sci* 2022;**31**:118–28.

38. Huerta-Cepas J, Szklarczyk D, Heller D *et al.* EggNOG 5.0: a hierarchical, functionally and phylogenetically annotated orthology resource based on 5090 organisms and 2502 viruses. *Nucleic Acids Res* 2019;**47**:D309–14.

39. Moriya Y, Itoh M, Okuda S *et al*. KAAS: an automatic genome annotation and pathway reconstruction server. *Nucleic Acids Res* 2007;**35**:W182–5.

40. Chen C, Wu Y, Li J *et al*. TBtools-II: a “one for all, all for one” bioinformatics platform for biological big-data mining. *Mol Plant* 2023;**16**:1733–42.

41. Li W, Godzik A. Cd-hit: a fast program for clustering and comparing large sets of protein or nucleotide sequences. *Bioinformatics* 2006;**22**:1658–9.

42. Mirdita M, Steinegger M, Breitwieser F *et al.* Fast and sensitive taxonomic assignment to metagenomic contigs. *Bioinformatics* 2021;**37**: 3029–31.

43. Morimoto Y, Uesaka K, Fujita Y, *et al*. A nitrogenase-like enzyme is involved in the novel anaerobic assimilation pathway of a sulfonate, isethionate, in the photosynthetic bacterium *Rhodobacter capsulatus*. *mSphere* 2024;**9**:e00498-24.

44. McCutcheon JP, Moran NA. Extreme genome reduction in symbiotic bacteria. *Nat Rev Microbiol* 2012;**10**:13–26.

45. Margulis L, Hinkle G. Large symbiotic spirochetes: *Clevelandina*, *Cristispira*, *Diplocalyx*, *Hollandina*, and *Pillotina*. In: *The Prokaryotes*. 1992, 3965–78.

46. Nishimura Y, Otagiri M, Yuki M *et al*. Division of functional roles for termite gut protists revealed by single-cell transcriptomes. *ISME J* 2020;**14**:2449–60.

47. Treitli SC, Kolisko M, Husník F *et al*. Revealing the metabolic capacity of *Streblomastix strix* and its bacterial symbionts using single-cell metagenomics. *Proc Natl Acad Sci USA* 2019;**116**:19675–84.

48. Raymond J, Siefert JL, Staples CR *et al*. The natural history of nitrogen fixation. *Mol Biol Evol* 2004;**21**:541–54.

49. McBride MJ. *Bacteroidetes* gliding motility and the type IX secretion system. *Microbiol Spectr* 2019;**7**:10.1128/microbiolspec.psib-0002-2018.

50. Paillat M, Silva IL, Cascales E *et al*. A journey with type IX secretion system effectors: selection, transport, processing and activities. *Microbiology* 2023;**169**:e001320.

51. Slakeski N, Seers CA, Ng K *et al*. C-terminal domain residues important for secretion and attachment of RgpB in *Porphyromonas gingivalis*. *J Bacteriol* 2011;**193**:132–42.
