# Supplementary figures and images for: Functional division of labor in motility, lignocellulose digestion, and nitrogen metabolism revealed for the Mixotricha paradoxa holobiont
Source: ISME J. 2025 Aug 20;19(1):wraf178. doi: 10.1093/ismejo/wraf178 (PMC12483993; doi:10.1093/ismejo/wraf178)

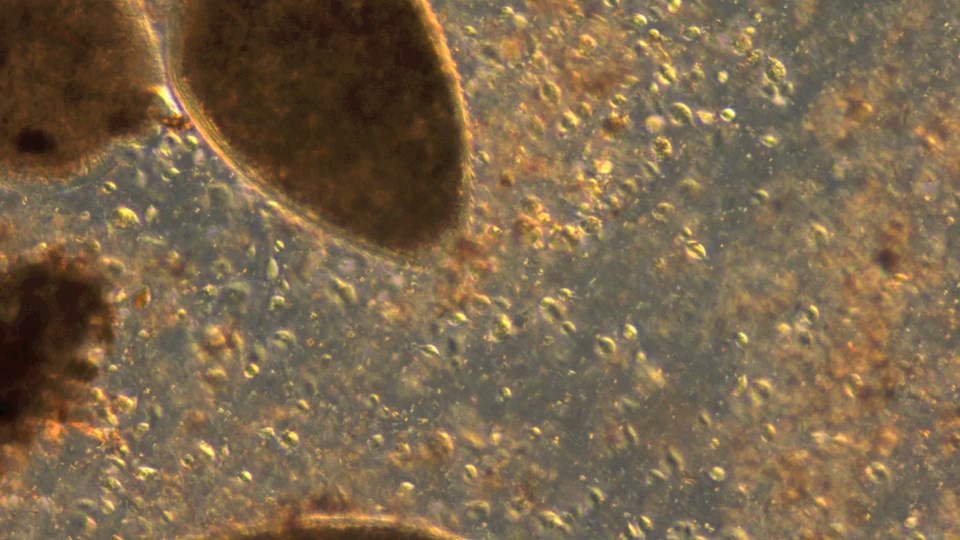

Supplement: Video_S1_still_image_wraf178 [file video_s1_still_image_wraf178.jpeg]

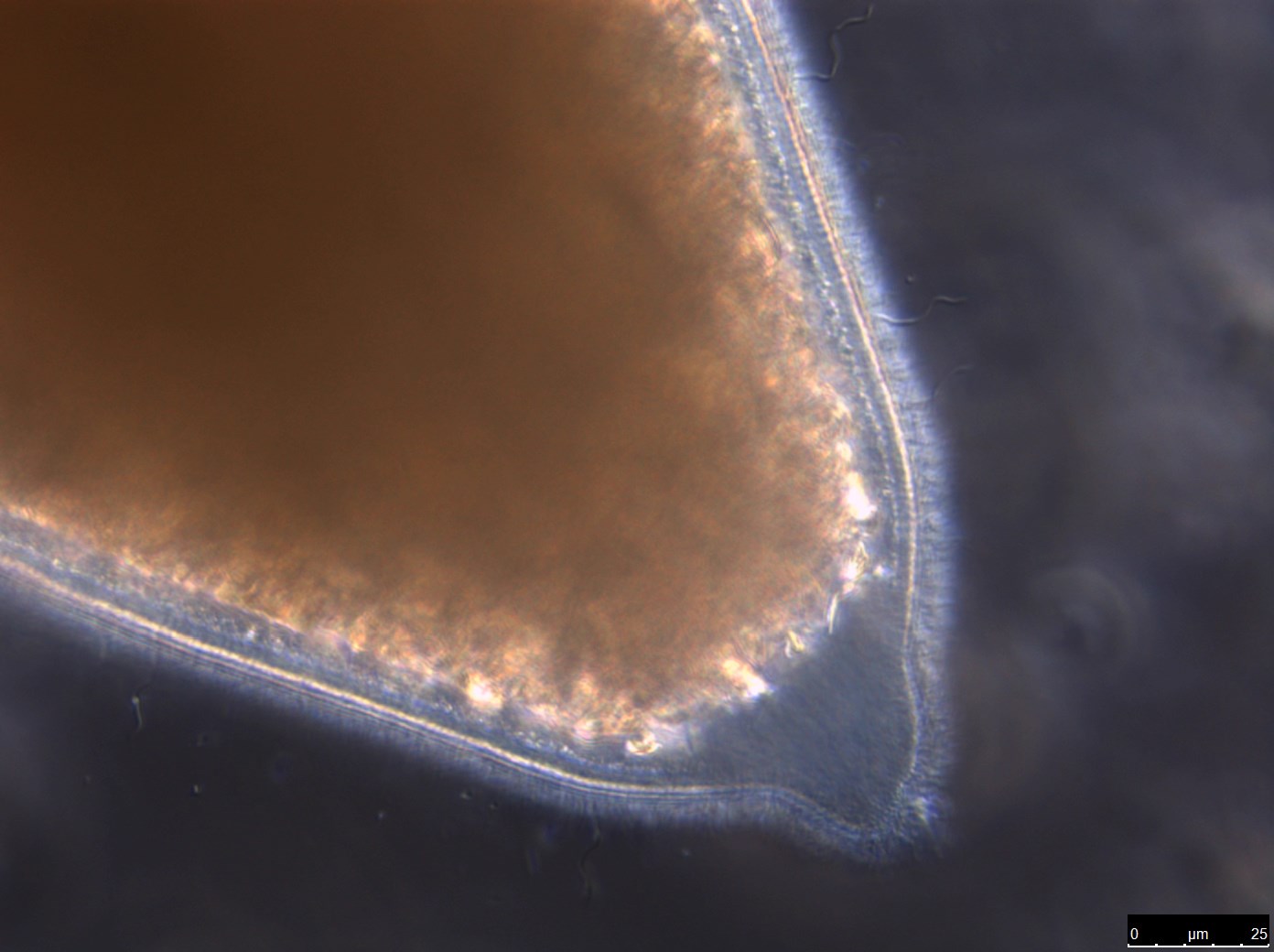

Supplement: Video_S2_still_image_wraf178 [file video_s2_still_image_wraf178.jpeg]
